# Towards an ecological understanding of readiness to engage with interventions for children exposed to domestic violence and abuse: Systematic review and qualitative synthesis of perspectives of children, parents and practitioners

**DOI:** 10.1111/hsc.12587

**Published:** 2018-07-10

**Authors:** Emma Howarth, Theresa HM Moore, Nicky Stanley, Harriet L. MacMillan, Gene Feder, Alison Shaw

**Affiliations:** ^1^ National Institute of Health Research (NIHR) Collaboration for Leadership in Applied Health Care (CLAHRC) East of England, Cambridge Institute of Public Health University of Cambridge Cambridge UK; ^2^ Population Health Sciences, Bristol Medical School University of Bristol Bristol UK; ^3^ NIHR Collaboration for Leadership in Applied Health Care (CLAHRC) West Whitefriars UK; ^4^ School of Social Work, Care and Community University of Central Lancashire Preston UK; ^5^ Faculty of Health Sciences McMaster University Hamilton Ontario Canada; ^6^ Centre for Academic Primary Care, Population Health Sciences, Bristol Medical School University of Bristol Bristol UK

**Keywords:** children, domestic violence, ecological framework, intervention, qualitative synthesis, readiness

## Abstract

Children who grow up in homes affected by domestic violence and abuse (DVA) are at risk of poor outcomes across the lifespan, yet there is limited evidence on the acceptability and effectiveness of interventions for them. A recent review of child‐focused interventions highlighted a gap in understanding the factors influencing the willingness of parents and children to engage with these programmes. We conducted a systematic review of qualitative evidence on the experiences of receiving and delivering interventions with the aim of identifying factors at different levels of the social–ecological context that may influence parent and child readiness to take up interventions. We searched literature till April 2016 and found 12 reports of eight programmes. Two authors independently screened papers for inclusion, extracted data and identified the first‐ and second‐order constructs. The third‐order constructs were derived and fitted to the ecological framework to inform a picture of readiness to engage with interventions. Three key findings emerged from this review: (a) parent and child readiness is influenced by a complex interplay of individual, relationship and organisational factors, highlighting that individual readiness to take up child‐focussed interventions must be viewed in an ecological context; (b) the specific process through which women become ready to engage in or facilitate child‐focussed interventions may differ from that related to uptake of safety‐promoting behaviours and requires parents to be aware of the impact of DVA on children and to focus on children's needs; (c) there are distinct but interlinked processes through which parents and children reach a point of readiness to engage in an interventions aimed at improving child outcomes. We discuss the implications of these findings for both practice and research.

**What is known about this topic**

- Little evidence exists on readiness of parents and children affected by DVA to take up “child‐focussed” interventions.
- Studies show practitioner, organisational and program‐related factors can affect family engagement in child‐oriented programmes.
- Viewing readiness through an ecological perspective can elucidate multilevel influences that affect willingness to participate in an intervention.

**What this paper adds**

- An ecological framework is helpful for understanding the complex interplay of individual, relational, situational and organisational factors that influence parent and child readiness.
- Distinct but interlinked processes emerge around their readiness to engage.
- A range of modifiable factors can be addressed to increase parent and child readiness; efforts to enhance readiness must extend beyond a focus on individual factors.

## 1 **INTRODUCTION**

### 1.1 Scale and impact of children's exposure to domestic violence and abuse

Domestic violence and abuse (DVA) is a gendered problem representing one of the greatest health risks for women and children (Feder & Potter, 2017; Hegarty et al., 2010; WHO, 2002). A United Kingdom prevalence study found that 15% of children had witnessed at least one form of DVA at some point during childhood and 3% of those have witnessed an incident in the last year (Radford et al., 2011). Similar prevalence rates are reported in US and Canadian population surveys (Afifi et al., 2014; Finkelhor, Turner, Shattuck, & Hamby, 2013).

Exposure to DVA is associated with a significant risk to children's physical and psychological safety and well‐being across the lifespan (Evans, Davies, & DiLillo, 2008; Howell, Miller, Lilly, & Graham‐Bermann, 2013; Wolfe, Crooks, Lee, McIntyre‐Smith, & Jaffe, 2003) and an increased risk of experiencing other forms of maltreatment (Hamby, Finkelhor, Turner, & Ormrod, 2010). Childhood exposure to DVA is associated with an increase in internalising symptoms (e.g. anxiety, depression), externalising behaviours (e.g. aggression) and trauma symptoms (Evans et al., 2008; Kitzmann, Gaylord, Holt, & Kenny, 2003; Wolfe et al., 2003). It is also associated with negative outcomes in adulthood, including mental health problems and criminal behaviour, in addition to DVA victimisation and perpetration. There is considerable evidence to suggest that negative outcomes in adulthood are mediated by adjustment difficulties, particularly behaviour problems that develop during childhood, including adolescence (Cui, Durtschi, Donnellan, Lorenz, & Conger, 2010; Fergusson & Horwood, 1998; Lussier, Farrington, & Moffitt, 2009; Russell, Springer, & Greenfield, 2010). It is therefore critical to offer effective interventions in childhood to ameliorate the risk of negative outcomes in childhood, adolescence and adulthood.

### 1.2 Interventions for children exposed to DVA

Interventions designed to prevent or reduce mental health difficulties following children's exposure to DVA were developed (British Columbia Centre of Excellence for Women's Health, 2013; Howarth et al., 2015; Rizo, Macy, Ermentrout, & Johns, 2011). However, evidence of their effectiveness and acceptability is limited. Our recent mixed method evidence synthesis identified evidence gaps, highlighting the need for better quality evaluative studies. One such gap was the limited consideration of psychological readiness of children and parents to engage in child‐focussed interventions and how this may impact on the acceptability of interventions and treatment outcomes (Howarth et al., 2015).

Many interventions that explicitly aim to enhance outcomes for children exposed to DVA require participation of both the victimised parent and child (Howarth, Moore, et al., 2016; Katz, 2015). Even where parents’ direct involvement is not required, it is often incumbent upon parents to facilitate their children's access to interventions and to engage with the questions and challenging behaviour that may result from children's increased awareness of abuse and attempts to process their experiences (Humphreys, Mullender, Thiara, & Skamballis, 2006). Offering interventions to the children of parents who are not fully engaged in their child's therapeutic journey may run the risk of alienating parents from supportive services altogether. It is potentially unethical to withhold services from a child who could benefit simply because a parent is unwilling to support engagement. Indeed, a UK study emphasised the importance of enabling children to access interventions independently of their parent (Itzin, Taket, & Barter‐Godfrey, 2010). Similarly, encouraging a child to participate in an intervention before they are suitably ready could be extremely detrimental to their adjustment. With this in mind, understanding readiness as a potential modifier of willingness to engage in any intervention and as a determinant of children's and parents’ preferences for different types of interventions (at different stages of readiness) is important to enhance the understanding of which options are appropriate for children and parents at different points in the recovery process.

### 1.3 Readiness to engage in DVA interventions

The term *readiness* in relation to therapeutic interventions refers to a person's willingness to change their behaviour and/or engage in an intervention. However, where DVA is concerned, another party is responsible for the problem behaviour (Prochaska et al., 1994). Thus, the term readiness is often used in relation to the abused party's willingness to take steps to reduce an exposure to DVA or to enhance well‐being, regardless of the actual outcomes (Cluss et al., 2006).

Several process models have been developed to elucidate the factors that may facilitate or inhibit change for women experiencing DVA. Commonly, these models conceptualise readiness as a process rather than a dichotomous state and acknowledge that different types of intervention may be required at different points along a help‐seeking and recovery trajectory. This is supported by women's accounts of the type of support that is valued, which may differ according to readiness (Feder, Hutson, Ramsay, & Taket, 2006).

The development of DVA interventions using the transtheoretical model (TTM) of behaviour change (Prochaska & DiClemente, 1992) has been proposed, adapting the model to reflect the readiness of women affected by DVA (Frasier, Slatt, Kowlowitz, & Glowa, 2001; Haggerty & Goodman, 2003; Hindmarsh, Knowlden, McMurchie, Schofield, & Hegarty, 1998; Zink, Elder, Jacobson, & Klostermann, 2004). Despite adaptations positioning women at the centre of the intervention (Hegarty, O'Doherty, Gunn, Pierce, & Taft, 2008), criticisms persist about the use of the TTM with DVA that TTM considers the behaviour of only one actor—the woman—and not the that of perpetrator. The autonomous *readiness* of the woman may not be the main precursor to change. The TTM model requires a target for change and for victims of DVA; it is not always clear what that should be. For example, leaving an abusive relationship is often perceived as the target, but this can lead to increased danger for women (Campbell, Webster, et al., 2003; Nicolaidis et al., 2003; Stanley, Miller, Richardson Foster, & Thomson, 2011). Finally, women affected by DVA may not proceed through each step of the TTM in order and may skip entire steps (Haggerty & Goodman, 2003).

Cluss et al. (2006) propose instead a psychological readiness model which describes the dynamic interplay of internal and external factors along a continuum of readiness to change, anchored at one end by maintaining the status quo and at the other with a desire for action. The model posits that the mobilisation of three internal factors—awareness, self‐efficacy and perceived support—determines an individual's position on the readiness continuum. Only when all three factors are aligned towards readiness, will an individual be in a position for action. The model highlights that readiness is also moderated by external factors such as economic resources, number of children and the characteristics of the organisations and professionals delivering support, which is in line with evidence on mental health interventions (Ingoldsby, 2010).

Given the range of factors that may shape readiness, an ecological framework—one that delineates the multilevel influences on a particular phenomenon—may be useful for conceptualising and organising the factors that may influence readiness to engage in DVA interventions. The ecological framework was first developed by Bronfenbrenner to explain how the inherent qualities of a child and his or her environment interact to influence development (Bronfenbrenner, 1977). Bronfenbrenner conceived of a person's environment comprised of five different nested levels: the microsystem (immediate environment in which a child lives and interacts), the mesosystem (interaction between different microsystems), the exosystem (social structures that indirectly affect the child), the macrosystem (culture) and the chronosystem (environmental events and transitions over the life course). This model was later extended by Belsky to acknowledge the effect of individual developmental factors, such as personality, on a persons’ interaction with the microsystem and other levels of the socio–ecological context (Belsky, 1980)

Ecological models have been widely used in other fields, such as public health, mental health and responses to sexual violence (Golden & Earp, 2012; Stormshak et al., 2011). The framework has also been used as a heuristic tool to delineate factors that are predictive of gender‐based violence at different levels of the social ecology (Heise, 2011; Hindmarsh et al., 1998). As of yet the ecological framework has not been used as a tool to identify and organise the range of factors—from individual to system level—that may impact on the decisions of parents and children to engage with interventions designed to improve child outcomes following exposure to DVA.

### 1.4 Current study

This paper draws on a systematic review and synthesis of qualitative evidence on child, parent and practitioner experiences of receiving and delivering interventions for children exposed to DVA, with the aim of identifying factors at various levels of the social–ecological context that contribute to readiness to engage with child‐focussed DVA interventions. The overarching aim of this review was to improve the understanding of how uptake can be enhanced in future interventions through targeting modifiable factors that influence parent and child readiness. We sought to answer the following questions: (a) what are the individual, relational and organisational factors that influence parents and children's readiness to engage with child‐focussed DVA interventions? (b) How does readiness of a parent and child interact to influence willingness of both parties to participate in an intervention?

## 2 METHOD

During an evidence synthesis, the “IMPROVE” review, examining the effectiveness, cost‐effectiveness and acceptability of interventions for children exposed to DVA (Howarth et al., 2016), we reviewed qualitative literature on the perspectives of children, parents and providers about interventions. We report on a synthesis of this qualitative evidence using an interpretive perspective in which we identified constructs in the original papers, compared these across studies and generated new overarching constructs that accounted for and further elaborated all those in the included papers. Adding the synthesis team's interpretation, we wrote a synthesis of these overarching constructs that conveyed readiness to engage with interventions shaped by an ecological perspective (see protocol Moore, Howarth, Feder, & Heawood, 2013).

### 2.1 Search methods

We searched MEDLINE, PsycINFO and EMBASE on OVIDSP; CINAHL on EBSCO; the Cochrane Central Database of Controlled Trials (CENTRAL) on the Cochrane Library; the Science Citation Index; Social Science Citation Index on Web of Science; the Applied Social Science and Abstracts Index (ASSIA); International Bibliography of the Social Sciences (IBSS); Social Services Abstracts; Sociological Abstracts on ProQuest; Social Care Online; the WHO trials portal and clinical trials.gov from inception to April 2016. We did not limit the search by study design, date or language. Letters, editorials and records with no abstract were excluded. We used MeSH and text word terms for <Children and adolescents> combined with terms for <DVA> and combined these with text word terms for <exposure of children to domestic violence or witnessing or growing up with domestic violence>. Search details are listed in the supplementary material.

### 2.2 Study selection, appraisal and data extraction

Two reviewers (TM, EH or AS) independently screened titles, abstracts and full‐text papers against the inclusion criteria. Any discrepancies between reviewers were resolved though discussion with recourse to a third reviewer GF. Following previous qualitative syntheses (Campbell, Pound, et al., 2003a; Malpass et al., 2009), we applied two initial screening criteria/questions to each paper to determine: *Is this qualitative research*? And *is this paper relevant to the synthesis*? Included papers needed to be peer reviewed and to report qualitative data from children or parents who had experienced an intervention or practitioners who had provided an intervention for children exposed to DVA. We also included author interpretations derived from such qualitative data.

We extracted the study details from each paper (Table 1 and see Supporting Information Table S1. We then extracted verbatim quotes from children, parents or practitioners (including field notes from observations) regarding their experiences of interventions (first‐order constructs) and authors’ interpretations of the qualitative data from the study participants (second‐order constructs). Each included paper was independently appraised by two reviewers using the Critical Appraisal Skills Program (CASP) checklist for qualitative research (CASP, 2010).

**Table 1:** Summary of study details

Programme	Study ID Country	Respondents	Intervention	Delivered to	Setting	Programme	Characteristics of children	Data collection	Method of analysis
1	Paris (1999), USA	Children *n* = 1g *n*=3b Parents (both) *n*=8m, *n*=6f Practitioners *n*=14	Group parallel psychoeducation for parents and children	Children, both parents, parallel separate sessions—some conjoint parental and familial sessions. Male respondents were court mandated	University of Florida campus	RSVP (Responsible Steps Towards Violence Prevention) Programme and BIP (Batterers intervention programme)	7–8 years n=3 12 years n=1	Focus groups	Constant comparison
2	Peled and Edleson (1992), USA	Children n=30Parents (n=16m, n=5f)Practitioner n=9	Group parallel psychoeducation for parents and children	Children, mothers. Some fathers. Parallel separate sessions. Some fathers attended a court mandated programme for perpetrators of abuse	Community DV services	Parents DAP (Domestic abuse project of Minneapolis)	4–12 years n=30	Interviews and observation	Naturalistic research paradigm inductive content analysis
	Peled (1998), USA	Children n=14Mothers n=12	As above	As above	As above	As above	Mean age 11.3 (10–13 years)	Interviews	Inductive content analysis. Phenom‐ecological inquiry. Naturalistic enquiry
	Peled and Edleson (1999), USA	Parents n= 64mn=41f	As above	As above	As above	As above	Mean age 10.4 years. (4–18 years)	Structured telephone interview	Inductive content analysis
3	Humphreys et al. (2006), UK	Mothers n=45	Activities to improve mother–child communication	Mother & child (dyads)	Refuge	“Talking with my Mum”	Children age 5–16 years	Focus groups	Action research
	Humphreys et al. (2011), UK	Children n= 25 g n=27bMothers n=45Practitioners (refuge workers) n=15	As above	As above	As above	As above	As above	As above	Action research. Grounded research theory
4	Kearney and Cushing (2012), USA	Mothers n=5Practitioners (therapists)^*^	Group psychoeducation	Mothers with children attending therapy (child intervention not described)	Community DV services	Maternal group psychoeducation for children receiving therapy	5–12 years	Interviews	Not described
5	Thompson (2009), USA	Practitioner (therapist/author) n=1	Child play therapy	Children small group	School	Play therapy	6–7 years	Participant observation	Erickson's analytic induction. Linking statements about the data based on evidentiary warrant
	Thompson (2011), USA	As above	As above	As above	As above	As above	As above	As above	As above
6	Jarman (2014), UK	Children n=4b	Drama therapy	Boys	Not stated	Group drama therapy based on embodiment, projection and role (EPR) model	7–9 years	Interviews and observation	Thematic analysis
7	Ermentrout et al. (2014), USA	Children n=8 Mothers n=18 Practitioners n=7	Group support	Mothers and children. Parallel separate sessions. Mothers were/had been in DVA relationships and were defendants in criminal justice system	Community	MOVE Mothers Overcoming Violence through Education and Empowerment. Group parenting and psychoeducation	No data but range included children both below 7 years and over 7 years of age	Interviews and focus groups	Thematic analysis. Constant comparison
8	Cater (2014), Sweden	Children n=16	Individual talking therapy	Children	Community	Trappan‐modellen (staircase) guided therapy. Guidelines	4–19 years	Interviews	Thematic analysis. Constant comparison

Notes

b: boys; f: fathers; g: girls; m: mothers.

*Number not stated.

### 2.3 Synthesis process

Having identified the first‐ and second‐order constructs, we compared these across the papers, examining them for convergence and divergence in language and meaning, in order to generate overarching third‐order constructs (interpretations of the synthesis team) that encompassed and further elaborated all constructs in the original papers.

All the first‐ and second‐order constructs were entered into a table for each paper within each respondent group (child, parent and practitioners). The first‐ and second‐order constructs were then compared across papers. This process enabled us to develop a table of overarching third‐order constructs for each participant group (see Supporting Information Table S2).

Finally, we synthesised the third‐order constructs for each participant group, before integrating the data for all participant groups relevant to each third‐order construct. During this process, we related the synthesis of the third‐order constructs to three levels of the ecological framework (personal, interpersonal and context), keeping track of the participant group (child, parent and practitioner) from which the data were derived (Tables 3, 4, 5, 6).

## 3 RESULTS

### 3.1 Search

**Figure 1:**
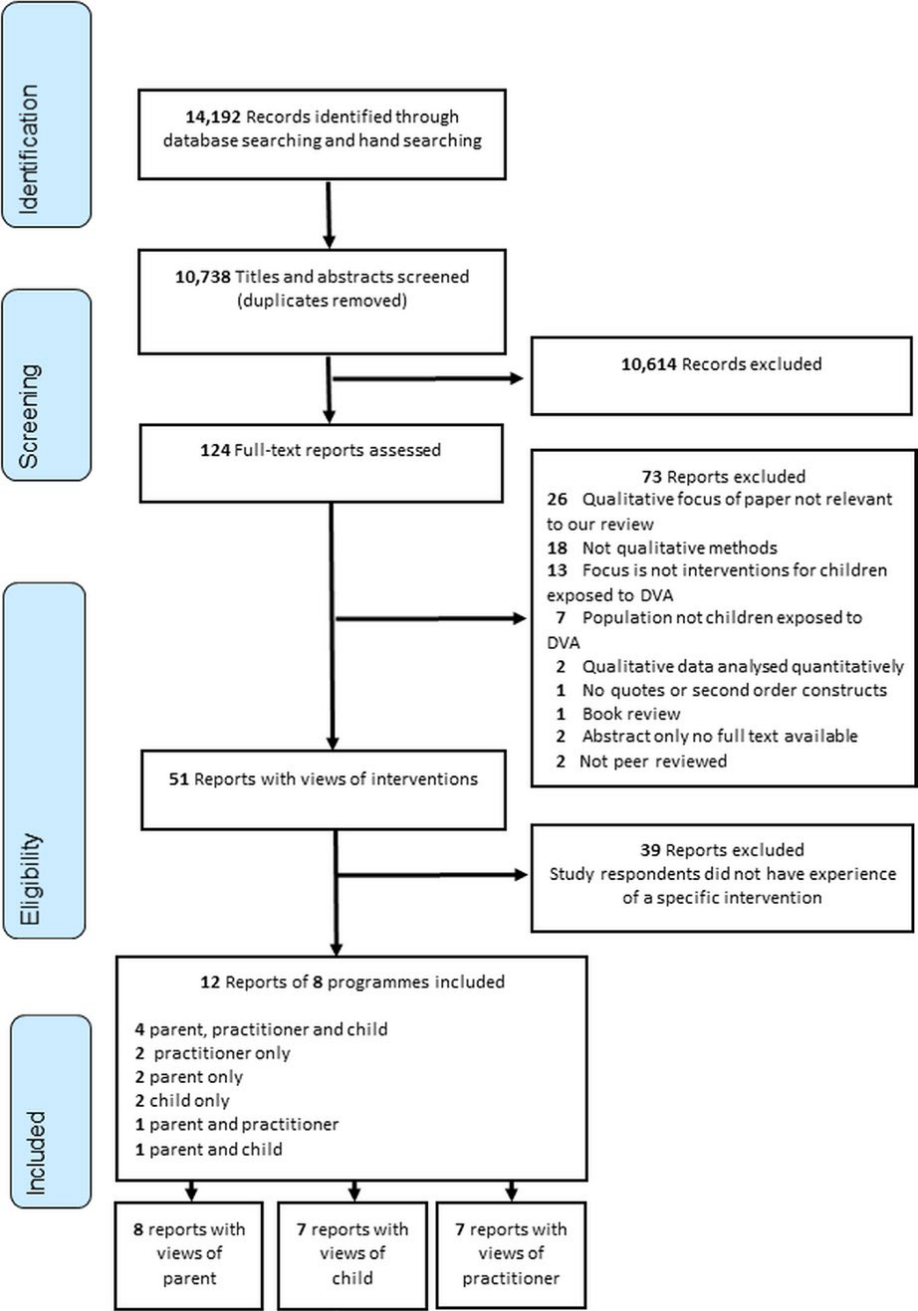
Flow of studies through the review

We identified 10,783 references (Figure 1). Reading titles and abstracts produced 124 potentially relevant references, for which we obtained full‐text copies. We excluded 73 papers that did not meet our inclusion criteria. Of the 51 remaining papers, 39 were excluded as they did not report participants’ experience of an intervention. Our final sample comprised 12 papers reporting on eight interventions (Table 1 see Supporting Information Table S1).

### 3.2 Quality of the papers

Eight of the 12 papers were consistently rated as *good quality* by the reviewers, each scoring 10 on the CASP checklist (See Table 2) (Ermentrout, Rizo, & Macy, 2014; Humphreys, Thiara, & Skamballis, 2011; Paris, 1999; Peled, 1998; Peled & Edleson, 1999, 1992; Thompson, 2009, 2011). Three scored between 7 and 9, (Cater, 2014; Humphreys et al., 2006; Kearney & Cushing, 2012) suggesting all were good or acceptable quality. One scored 5 (Jarman, 2014). A lack of space to report methods, rather than a lack of research quality, seemed to be the reason for lower quality scores. A sensitivity analysis indicated that the removal of the four “weaker” papers did not change the main synthesis findings.

**Table 2:** Quality markers of each paper as measured with CASP

CASP Item	Cater (2014)	Jarman (2014)	Ermentrout et al. (2014)	Humphreys et al. (2006)	Humphreys et al. (2011)	Kearney and Cushing (2012)	Paris (1999)	Peled and Edleson (1992)	Peled (1998)	Peled and Edleson (1999)	Thompson (2009)	Thompson (2011)
Total *n*= “Yes”	9	5	10	8	10	7	10	10	10	10	10	10
Total *n*= “No”	0	1	0	2	0	2	0	0	0	3	0	0
Total *n*=“Can't tell”	1	3	0	0	0	1	0	0	0	0	0	0
How valuable is the research to this meta‐synthesis? (Key, useful, marginal, not relevant)	Key	Useful	Key	Key	Key	Useful/Marginal^a^	Useful	Key	Key	Key	Useful	Useful
1 Was there a clear statement of AIMS of the research?	Yes	Yes	Yes	No	Yes	Yes	Yes	Yes	Yes	Yes	Yes	Yes
2 Is a qualitative methodology appropriate?	Yes	Yes	Yes	Yes	Yes	Yes	Yes	Yes	Yes	Yes	Yes	Yes
3 Was the research design appropriate to address the aims of the research?	Yes	Can't tell	Yes	Yes	Yes	Yes	Yes	Yes	Yes	Yes	Yes	Yes
4 Was the recruitment strategy appropriate to the aims of the research?	Yes	Can't tell	Yes	Yes	Yes	Yes	Yes	Yes	Yes	Yes	Yes	Yes
5 Was the data collected in a way that addresses the research issue?	Yes	Yes	Yes	Yes	Yes	Can't tell	Yes	Yes	Yes	Yes	Yes	Yes
6 Has the relationship between researcher and participants been adequately considered?	Can't tell	Can't tell	Yes	Yes	Yes	No	Yes	Yes	Yes	No	Yes	Yes
7 Have ethical issues been taken into consideration?	Yes	Can't tell	Yes	Yes	Yes	Yes	Yes	Yes	Yes	No	Yes	Yes^b^
8 Was the data analysis sufficiently rigorous?	Yes	Adequate	Yes	Yes	Yes	No	Yes	Yes	Yes	No	Yes	Yes
9 Is there a clear statement of findings?	Yes	Yes	Yes	No	Yes	Yes	Yes	Yes	Yes	Yes	Yes	Yes
10 How valuable is the research?^c^	Yes	Yes	Yes	Yes	Yes	Yes	Yes	Yes	Yes	Yes	Yes	Yes

^a^Highly relevant to our review but uncertain quality because of lack of reporting in paper.

^b^Retrospective analysis. No mention of formal ethical review, but it is clear that ethical considerations were taken into account during the group therapy.

^c^Did the researchers discuss the contribution of the study to existing knowledge or understanding, for example, do they consider the findings in relation to current practice or policy or relevant research‐based literature? Do they identify new areas where research is necessary? Have the researchers discussed whether or how the findings can be transferred to other populations or considered other ways the research may be used? Is the research useful to this synthesis?

**Table 3:** The third‐order constructs for the individual level of the ecological model: Personal factors shaping children's readiness to engage. Source material from all papers and high quality papers

Respondent	Third‐order constructs	All papers	High‐“quality” papers
Initiation of engagement			
Children	Self‐motivation, recognition of own needs	Cater (2014)	Cater (2014)
Understanding and acknowledgement of DVA			
Children might not understand that DVA is the root cause of their involvement with an intervention			
Children	Adjustment to the “new reality” in their lives	Peled (1998)	Peled (1998)
Children	Acknowledging DVA had been a part of their lives	Peled (1998) Jarman (2014)	Peled (1998)
Children	DVA, and engagement with a DVA‐focused intervention, may be of marginal concern to children who may be engaged with other normal developmental milestones or other immediate family traumas	Peled (1998) Thompson (2011)	Peled (1998) Thompson (2011)
Children	Learning the violence vocabulary	Paris (1999) Peled and Edleson (1992)	Paris (1999) Peled and Edleson (1992)
Children	Defining or labelling abuse	Peled and Edleson (1992) Paris (1999) Peled (1998)	Peled and Edleson (1992) Paris (1999) Peled (1998)
Children	Attribution of responsibility for DVA	Peled (1998) Peled and Edleson (1992) Jarman (2014)	Peled (1998) Peled and Edleson (1992)
Children	Recognising equality of gender roles	Jarman (2014)	
Recognising the effect of DVA on mothers and other children and having empathy			
Children	Empathy for mother's situation	Peled (1998)	Peled (1998)
Children	Recognising their mother's strength	Jarman (2014)	
Children	Development of empathy	Thompson (2011)	Thompson (2011)
Breaking the secret			
Children	Helping mothers and other children [altruism as motivation for joining in]	Humphreys et al. (2011)	Humphreys et al. (2011)
Breaking the secret was difficult for children/children did not want to talk about the past or their fathers. Children felt their situation to be shameful/the presence of nonabusive parent (mother) helped children with the activities			
Children	Not wanting to talk about the past/violence Not wanting to talk about their fathers	Humphreys et al. (2011) Peled (1998) Jarman (2013) Cater (2014)	Humphreys et al. (2011) Peled (1998) Cater (2014)
Children	Hesitancy to share what has happened	Thompson (2009) Paris (1999)	Thompson (2009) Paris (1999)
Children	Readiness to talk	Paris (1999) Peled and Edleson (1992) Thompson (2011) Thompson (2009)	Paris (1999) Peled and Edleson (1992) Thompson (2011) Thompson (2009)
Children	“I am not alone”: Beneficial; release of stress particularly beneficial to children who have not spoken of it before Reducing shame and guilt	Peled and Edleson (1992) Thompson (2011) Ermentrout et al. (2014)	Peled and Edleson (1992) Thompson (2011) Ermentrout et al. (2014)
Children	Child perception of benefits	Humphreys et al. (2011) Paris (1999) Jarman (2014)	Humphreys et al. (2011) Paris (1999) Jarman (2014)
Children	Building trust between practitioner and child, helps break the secret	Cater (2014)	Cater (2014)
Children	Therapeutic relationship can assist engagement	Cater (2014)	Cater (2014)
Children	Children must be motivated if intervention is to work	Cater (2014)	Cater (2014)
Readiness to “Break the secret”			
Children	Differential readiness between parents and children as a barrier to uptake	Cater (2014)	Cater (2014)
Children	Mothers do not understand child needs, older children use own initiative to seek help	Cater (2014)	Cater (2014)
Children	Initiation driven by adult assumption of child needs	Cater (2014)	Cater (2014)
Children	Children's participation may be aided by involving them in decision to take up a programme	Cater (2014)	Cater (2014)
Children	Child must be motivated if participation is to work	Cater (2014)	Cater (2014)

**Table 4:** The third‐order constructs for the individual level of the ecological model: Personal factors shaping readiness to engage of parents and practitioners. Source material from all papers and high‐quality papers

	Third‐order constructs	All papers	High‐“quality” papers
Understanding and acknowledgement of DVA			
Parents	Readiness of parents (fathers) to name and acknowledge DVA	Peled and Edleson (1999)	Peled and Edleson (1999)
Parents	Acknowledgement of negative impact of DVA on child	Humphreys et al. (2011) Kearney and Cushing (2012) Peled and Edleson (1999)	Humphreys et al. (2011) Peled and Edleson (1999)
Able to consider needs of their children			
Parents	Parents able to see beyond own needs to those of their child	Humphreys et al. (2011) Kearney and Cushing (2012) Peled and Edleson (1999) Ermentrout et al. (2014)	Humphreys et al. (2011) Peled and Edleson (1999) Ermentrout et al. (2014)
Parents	Mothers’ willingness to talk to their children about the past	Humphreys et al. (2011)	Humphreys et al. (2011)
Parents may fear children's disclosures			
Practitioners	Parents fear of children disclosing information	Ermentrout et al. (2014)	Ermentrout et al. (2014)
Practitioners	Parents may coach children not to talk	Ermentrout et al. (2014)	Ermentrout et al. (2014)
Differential readiness between parents and children as a barrier to uptake			
Parents	Mismatch in readiness; children are ready but mothers are not	Humphreys et al. (2011)	Humphreys et al. (2011)

**Table 5:** The third‐order constructs for the interpersonal level of the ecological model: Relational factors shaping readiness to engage (all respondents). Source material from all papers and high quality papers

Respondents	Third‐order constructs	All papers	High‐“quality” papers
The role of supportive relationships with practitioners in priming readiness to engage			
Practitioners	Facilitation of intervention (timing and readiness of mothers) by practitioners	Humphreys et al. (2011)	Humphreys et al. (2011)
Parents	Mothers feel that children are in a safe and confidential intervention/space/setting	Ermentrout et al. (2014)	Ermentrout et al. (2014)
Priming work might be needed			
Practitioners	Priming of mothers before introducing the intervention may be worthwhile. Priming or preparatory work with parents on the impact of DVA on their child. Priming children in advance of the intervention might be worthwhile	Humphreys et al. (2006) Peled and Edleson (1992) Ermentrout et al. (2014)	Peled and Edleson (1992) Ermentrout et al. (2014)
Parents	Interference by coparents may prevent child attending	Ermentrout et al. (2014)	Ermentrout et al. (2014)
Interpersonal trust between recipients			
Practitioners/children	Peer bonding gives support and aids dialogue	Paris (1999) Ermentrout et al. (2014) Thompson (2009) Thompson (2011)	Paris (1999) Ermentrout et al. (2014) Thompson (2009) Thompson (2011)
Interpersonal trust between recipients and providers of interventions			
Practitioners	Developing a shared understanding between the parent and child of their situations	Humphreys et al. (2011)	Humphreys et al. (2011)
Parents	“I am not alone”/learning from each other (comes through process of being in a group)	Kearney and Cushing (2012)	
Children	Hesitancy to share what has happened. Readiness to talk. Developing trust in the group. Children need time to develop trust in the group	Peled and Edleson (1992) Paris (1999) Thompson (2009) Thompson (2011) Cater (2014) Jarman (2014) Ermentrout et al. (2014)	Peled and Edleson (1992) Paris (1999) Thompson (2009) Thompson (2011) Cater (2014) Ermentrout et al. (2014)
Children	Being specifically asked (by counsellor) about violence was a key to opening up	Cater (2014)	Cater (2014)
Children	Child must trust the counsellor to participate	Cater (2014)	Cater (2014)
Children	Safe uninvolved adult was preferred	Cater (2014)	Cater (2014)
Power in child adult relationship may constrain child participation			
Parents	Parents (main carer) may prevent children attending intervention (Differential readiness)	Cater (2014)	Cater (2014)
Parents	Children may opt not to engage even if they are attending (in defiance of adult's wishes)	Cater (2014)	Cater (2014)
Practitioner	Interference by co‐parents may prevent child attending (Differential readiness)	Ermentrout et al. (2014)	Ermentrout et al. (2014)
Rules of the group helped children develop trust			
Children	Developing group norms and rules, for example, confidentiality “Ok not to talk” feeling supported in the group and not compelled to talk	Peled and Edleson (1992)	Peled and Edleson (1992)

**Table 6:** The third‐order constructs for the contextual level of the ecological model: External factors shaping readiness to engage (all respondents). Source material from all papers and high quality papers

Respondents	Third‐order constructs	All papers	High “quality” papers
Change in circumstances may require support			
Children	Adjustment to the “new reality” in their lives	Peled (1998) Thompson (2011)	Peled (1998) Thompson (2011)
Children	Living in a refuge/shelter	Peled (1998)	Peled (1998)
Shelter experience can provide stability as a base for change for families (mothers and children)			
Children	Living in a refuge/shelter	Peled (1998)	Peled (1998)
Practitioners	Situational readiness: families must not be in crisis	Humphreys et al. (2011) Humphreys et al. (2006)	Humphreys et al. (2011)
For families living in the community			
Parents	Overcoming practical barriers to attending	Ermentrout et al. (2014)	Ermentrout et al. (2014)
Practitioners’ readiness to deliver therapeutic child‐focused interventions depended on their skills			
Practitioners	Practitioners’ skills of working with women and children	Humphreys et al. (2011) Ermentrout et al. (2014)	Humphreys et al. (2011) Ermentrout et al. (2014)
Children	Ability of practitioner to adapt to the child's state of mind in each session	Cater (2014)	Cater (2014)
Intervention setting is important			
Parents	Mothers feel that children are in a safe and confidential intervention/space/setting	Ermentrout et al. (2014)	Ermentrout et al. (2014)
Flexibility/adaptability of the intervention itself			
Practitioners &Children	Adaptability of the intervention/flexibility of the practitioner helped practitioners to deliver it. And helped children engage	Ermentrout et al. (2014) Cater (2014)	Ermentrout et al. (2014) Cater (2014)
Organisational readiness to support engagement with and delivery of interventions			
Practitioners	Organisational readiness	Humphreys et al. (2011)	Humphreys et al. (2011)

### 3.3 Participants and intervention types

Detailed descriptions of the participants and interventions can be found in Table 1 (see Supporting Information Table S1). Seven papers reported children's views, seven parents’ views and eight practitioners’ views. Some papers reporting parents’ views included the views of abusive parents. In the included studies, all nonabusing parents were mothers and all abusing parents were fathers. The interventions included psychoeducation programmes, individual counselling, play therapy, drama therapy and guided self‐help. The children in the programmes were mostly aged between 4 and 12 years (Jarman, 2014; Kearney & Cushing, 2012; Paris, 1999; Peled, 1998; Peled & Edleson, 1992; Thompson, 2009, 2011) with two studies including children aged up to 16 years (Humphreys et al., 2006, 2011) or 19 years (Cater, 2014). In one study, mothers who were survivors of DVA were also involved with the criminal justice system (Ermentrout et al., 2014).

### 3.4 Synthesis

Results from the full synthesis identified constructs spanning the whole process of engaging with and receiving an intervention (Howarth, Moore, et al., 2016). Here, shaped by an ecological perspective, we consider factors which influence readiness to engage in an intervention at three levels: individual, interpersonal and contextual, from the viewpoint of children, parents and practitioners.

### 3.5 Individual factors shaping readiness to engage

#### 3.5.1 Children's personal readiness to engage

All papers reporting the experiences of children highlighted the importance of personal readiness for child engagement with interventions. The key dimensions were understanding and acknowledging DVA and willingness to break the secret of abuse.

Understanding and acknowledging DVA was an important part of children's readiness to take up an intervention. Children may not identify that DVA is happening in their lives (Jarman, 2014; Peled, 1998), and mothers’ responses to abusive situations—for example, moving to a refuge or shelter—may not always be accompanied by a change in children's understanding and awareness of DVA (Peled, 1998). Even if they recognise that DVA is present in their lives, children may not understand the impact on their mothers or be able to empathise with her. Moreover, children may not perceive the abuse to be the most important problem in their lives; other more immediate issues may occupy their attention, including “feelings of being rejected by their fathers” and “normal developmental challenges” (p. 420) (Peled, 1998). The relative “marginality” of DVA among the various challenges faced by a child may hinder their readiness to engage with an intervention.

Children's initial willingness to engage will sometimes be motivated by altruism and empathy. An appreciation of their mothers’ strength and a desire to support them can be the key features of children's motivation to attend an intervention (Jarman, 2014); in the studies we examined, children often expressed a desire to help their mothers or other children in the future, as reasons for starting an intervention (Humphreys et al., 2011). Conversely, some children seemed to have little voice in the decision to participate in an intervention, the decision being made by a parent or other related adult (Cater, 2014).This can make children's engagement problematic if they are not personally motivated to engage and feel that their needs have been assumed by adults (Cater, 2014).

At the start of an intervention programme, children may have limited ability to articulate their experiences of abuse. Through participation, they learn the language of abuse and how to talk about it as well as to develop the understanding that abuse is not acceptable or normal (Paris, 1999; Peled, 1998). As part of this process, children may begin to attribute responsibility for the abuse to the perpetrator and to understand that their father is to blame (Jarman, 2014). Stereotypical gender roles may also be addressed, with children understanding that violence is not an acceptable nor inevitable part of the male role (Jarman, 2014). Some need support to acknowledge that their fathers are responsible and to recognise the impact of DVA on their mother, themselves and any other children in the family. Through engaging in interventions, children gradually learn to identify types of abuse, which helps them to talk about, and process, their experiences (Paris, 1999; Peled, 1998; Peled & Edleson, 1992).

Building on a growing awareness of the nature of DVA, children have to reach a point where they are willing to “break the secret” of DVA as part of their readiness to fully engage with an intervention. Breaking the secret of DVA was consistently hard for children, whether they were personally motivated to attend an intervention or compelled to do so by a parent or professional (Cater, 2014; Ermentrout et al., 2014). They were often uncertain and hesitant about disclosing their experiences, as they felt their experiences to be shameful. In some instances, children were “coached” by a parent not to talk about the abuse, owing to parents’ fear of the consequences of children's disclosure, thus limiting their meaningful engagement. (Ermentrout et al., 2014).

Especially in the early stages of an intervention, children often did not want to talk about the past or their abusive parent (Cater, 2014; Humphreys et al., 2011). However, the presence of the nonabusive parent helped children's engagement, enabling them to understand the value of therapeutic activities and giving them “permission” to talk (Humphreys et al., 2011). Yet, children typically required time before they felt safe to share (Humphreys et al., 2011; Paris, 1999; Peled & Edleson, 1992; Thompson, 2009, 2011), often avoiding distressing topics such as their fathers or stepfathers during the early sessions of the intervention (Jarman, 2014).

Early engagement requires sensitive nurturing by those delivering interventions to encourage readiness to share; indeed, this in and of itself may be an important benefit of participation for children. Once children had begun to share the secret, talking about it was usually experienced as valuable and could bring a beneficial release of stress, particularly for children who had not spoken of it before. Sharing helped children to appreciate that they were not alone, and this in turn helped alleviate their sense of shame and guilt (Peled & Edleson, 1992; Thompson, 2011).

#### 3.5.2 Parents’ personal readiness to engage

For parents, readiness appears to be determined by the interplay of four internal factors: recognition of involvement in an abusive relationship, recognition of potential impact of DVA on children, the ability or willingness to see beyond their own needs to those of their children and to overcome their fear of what children might disclose. Where fathers were involved in decisions about child engagement, this related to interventions aimed at whole families. Those parents who doubted that an intervention was appropriate for their children were often those who also denied the abusive nature of their relationship:

*I wasn’t too involved with it. But I heard about how violence affects children. They never had a problem like that. Their mom is good with them. There was never violence at home. I went to DAP because there was some problems with my marriage. (p. 520 Father) (Peled & Edleson,* 1999 *)*

Where parents felt that their children had no need of an intervention, the family was less likely to engage. Parents who knew that their children had witnessed abuse were more likely to accept that an intervention might be desirable. Parents were unlikely to see the worth of an intervention if they believed that their children had no knowledge of the abuse, were too young to understand, did not directly witness it or perceived that DVA was insufficiently severe to place the children at risk of psychological harm. Parents tended to “grade” abuse their children had been exposed to and if they deemed it to be less severe (e.g. verbal abuse), they were less likely to want their children to engage in an intervention (Peled & Edleson, 1999). To be ready to engage in an intervention, parents needed to acknowledge that any form of exposure could have a detrimental impact on children's adjustment (Peled & Edleson, 1999). Although, for interventions involving groups of children, parents expressed fears about bringing their children into contact with others whom they perceived to have been exposed to more traumatic abuse (Peled & Edleson, 1999).

Parents also needed to be ready to look beyond their own needs to those of their children (Ermentrout et al., 2014; Humphreys et al., 2011; Kearney & Cushing, 2012; Peled & Edleson, 1999). Some acknowledged that they had been so absorbed in processing their own emotions and experiences that it had been difficult to devote time and emotion to offering or facilitating help for their children. In the words of one mother:

*I suppose if you'd have asked me eight months ago I might not have done it. Because I didn't want to accept myself what was going on. (p. 177 Mother) (Humphreys et al.,* 2011)

Moreover, as time elapses and their situation improves, parents may not see the need to access support for their children, particularly if children do not seem to be showing overt difficulties (Peled & Edleson, 1999).

Practitioners felt parents who were concerned about the effects of DVA on their children and who had noticed a need in their children were most motivated to seek help. Conversely, there were parents who did not wish to engage as the “preferred way to deal with the past was not to discuss it. Some saw it as a means of being protective of either themselves or their children” (p. 177) (Humphreys et al., 2011). As highlighted by practitioners, other parents hindered their children from attending as they were fearful of what children might reveal and concerned about the potential consequences of the children disclosing the abuse. This was true of both the abusive and nonabusive parent.

*There is this factor which is very real, which is the dads are worrying about what their children are coming here and saying. So I do think there’s some sabotage with the dads about the children participating and maybe even some fear on the moms’ part, “What will the kids say? What will they tell?” (p. 663) (Ermentrout et al.,* 2014)

#### 3.5.3 Differential readiness between parents and children as a barrier to uptake

In some instances, there may be a mismatch between parents and children with respect to the degree of readiness to engage in a child‐focussed intervention; one party may be ready to engage while the other may not, which may hinder family participation (Cater, 2014). One mother, while reluctant to discuss the effect of DVA (Humphreys et al., 2011), acknowledged that her daughters had been ready to talk about the abuse for some time, having already spoken about it to a special educational needs coordinator at their previous school. Supporting parents and children towards a similar point of readiness may therefore be a key in enhancing engagement with interventions. This is particularly important given that many of the interventions on offer are predicated on the involvement of both parents and children.

### 3.6 Relational factors shaping readiness to engage

Relational factors may impact readiness to engage in an intervention, particularly relationships between intervention providers and participants (whether parents or children) and between participants themselves (e.g. children in group interventions).

#### 3.6.1 The role of supportive relationships with practitioners in priming readiness to engage

Adapting to a new living situation (such as a shelter or move to a new home) in the aftermath of parental separation may require additional support from staff to help parents and children adjust to the new reality of everyday life, before they are ready to engage in a therapeutic intervention. Practitioners acknowledged their own key role in building supportive relationships with children and mothers and assisting them to engage with a programme. They described undertaking “priming” work with parents and children to prepare them to engage with an intervention, often undertaken alongside the provision of more practical support. Providing parents with information about why the intervention might be needed and how the DVA may be impacting their child(ren), as well as information about the content and processes of the intervention itself, is likely to increase its acceptability to potential participants and enhance willingness to participate. (Humphreys et al., 2006; Peled & Edleson, 1992).

#### 3.6.2 Interpersonal trust between recipients and providers of interventions

Children, parents and practitioners identified trust between those using and those delivering interventions as a crucial prerequisite for meaningful engagement. Trust between intervention providers and participants facilitates the process of sharing and aids participants’ readiness to talk, whether in the context of a group intervention or individual counselling (Cater, 2014; Jarman, 2014).Disclosure to a “safe” adult who is not a member of the child's family or a friend seems to be a key in helping children to discuss the abuse (Cater, 2014). Mothers also need to trust that their children, in attending an intervention, are in a safe and confidential space with trustworthy providers (Ermentrout et al., 2014).

For group‐based interventions, children also needed to build sufficient trust in other children to share their experiences (Paris, 1999; Peled & Edleson, 1992; Thompson, 2009, 2011). In this context, peer bonding can be a key for facilitating peer‐to‐peer support and for aiding dialogue between them (Ermentrout et al., 2014). In an intervention aimed at mothers and children, the process of hearing each other's experiences was an important part of developing a shared understanding of how the abuse was impacting different members of the family (Humphreys et al., 2011).

It takes time for children and parents to trust providers of interventions before they disclose experiences and feelings (Ermentrout et al., 2014; Thompson, 2011). Children need time to build this trust, and this process may be undermined if children are not actively involved in making the decision to participate and as a consequence are unwilling to engage in communication with intervention providers (Cater, 2014). Group rules initiated by intervention providers were important for facilitating trust through the creation of a “safe space” (Humphreys et al., 2006). These included rules about confidentiality: “whatever was said in the group, stays in the group” (p. 338) (Peled & Edleson, 1992).

### 3.7 Contextual factors shaping readiness to engage

**Figure 2:**
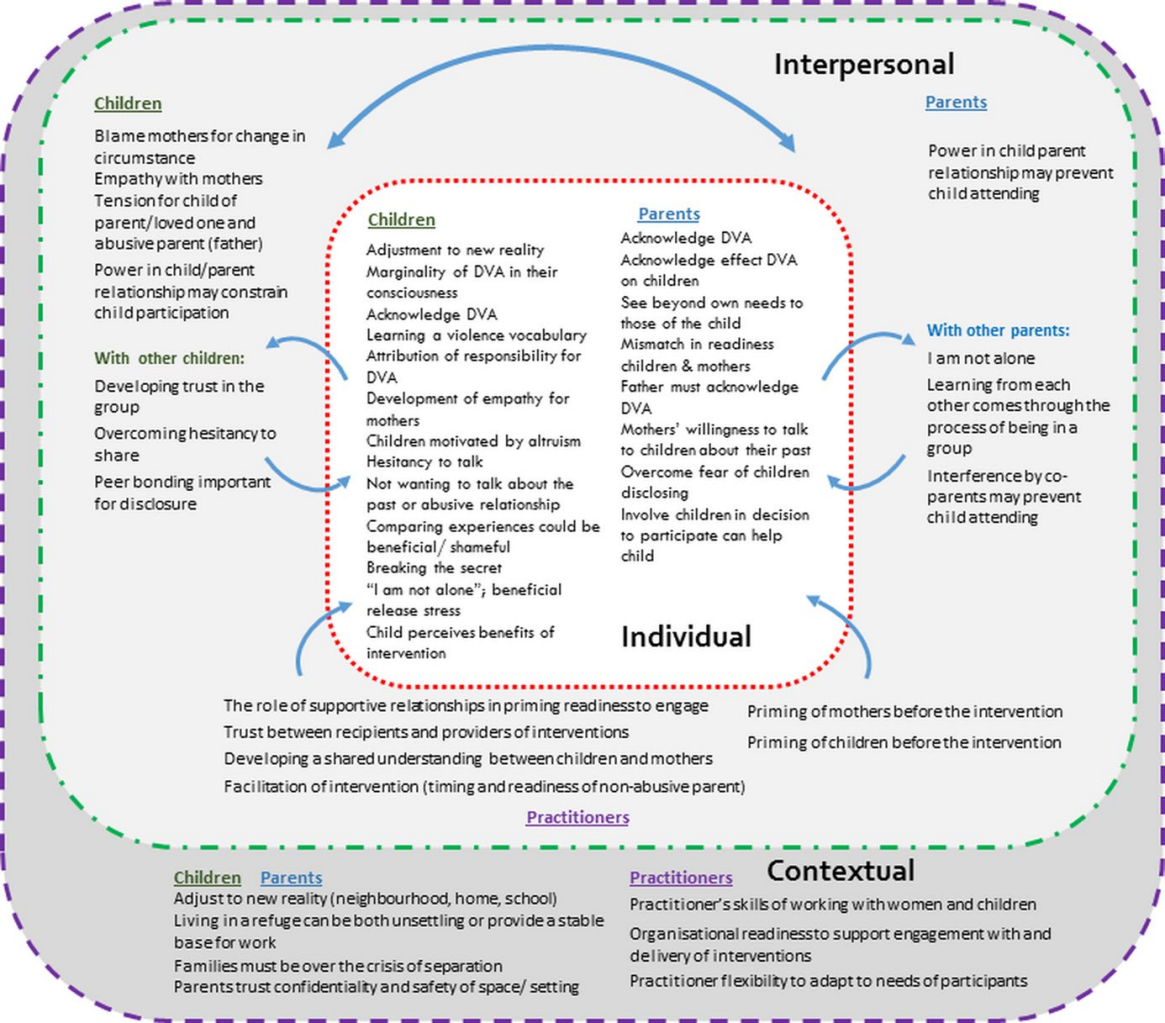
Personal, interpersonal and contextual themes as an ecological model, for parents, children and practitioners engaged in interventions for children exposed to domestic violence and abuse

External factors that may impact initial readiness to engage in interventions are aligned with the contextual level of the ecological model. Wider contextual factors, including the living situation of families impacted by DVA, may directly shape their readiness to engage, whereas organisational factors may indirectly influence readiness through practitioner readiness to deliver interventions (Figure 2).

#### 3.7.1 Change in living situations and readiness

Children‐accessing interventions following DVA have often experienced a significant change in their physical and material world as their family adjusts to a new reality. “The move implied a change of neighbourhoods, school, and general social environment” (p. 411) (Peled, 1998). Change goes together with a process of adaptation for children. For example, children who have moved to a shelter have to learn to adapt quickly to their new surroundings. (Peled, 1998). These contexts form the backdrop for children attending an intervention, and changes in their environment may be the key to their preparedness to participate. An important contextual aspect of parental readiness had the stability and time to engage with an intervention (Humphreys et al., 2006, 2011). Both in shelter settings and in the community, parents (particularly mothers) need to move towards a degree of stability in their home lives to have not only the emotional capacity but also the practical capacity to engage (Ermentrout et al., 2014; Humphreys et al., 2011). Practitioners were clear that a starting point for families’ safe and meaningful engagement with an intervention was a “situation of stability,” (p. 176) (Humphreys et al., 2011) which meant being beyond the crisis stage, separated from the perpetrator and being over the initial trauma of separation.

#### 3.7.2 Practitioners’ readiness to deliver therapeutic child‐focused interventions

To be willing to take up an intervention Mothers needed to have a degree of confidence in the setting and provider (Ermentrout et al., 2014). Practitioners delivering interventions needed to be ready to do so, which in turn was dependent on their personal characteristics, training, understanding of DVA and its impact and distinct from the skills required to deliver practical support. Practitioners need skills in working with women and children (Ermentrout et al., 2014; Humphreys et al., 2011) and the flexibility to be able to adapt an intervention to the specific needs of participants (Cater, 2014), for example, by responding to a child's state of mind in each session of an intervention and tailoring the material and communication appropriately. Ability to help women engage with an intervention could be limited by workers’ lack of understanding of mothers' trauma, or of why the mothers were not yet able to prioritise their children's needs (Humphreys et al., 2011).

#### 3.7.3 Organisational readiness to support engagement with and delivery of interventions

The theme of organisational readiness emerged from papers reporting an intervention delivered by refuge or shelter workers (Humphreys et al., 2006, 2011). From the perspective of practitioners, organisations already providing similar support (e.g. counselling) were seen as more ready and as having a better foundation upon which to deliver child‐focussed interventions. Organisations “in crisis” and facing challenges such as staff shortages were not considered ready to deliver interventions (Humphreys et al., 2011). Time pressures as well as lack of an appropriate skills base, both of which may be dictated by the organisational climate, have the potential to impede practitioner readiness to deliver interventions. The good will, enthusiasm, skills and resourcefulness of the workers, along with organisational support and training, were seen as key for organisational readiness, as was management “buy in” to the ethos of the intervention (Humphreys et al., 2011).

## 4 DISCUSSION

Previous research has focused mainly on the readiness of women who have experienced DVA to take up woman‐focussed interventions, with little attention to the process through which children reach a point of readiness to engage with interventions, or indeed the process through which parents become ready to support their children's recovery. The purpose of this review was to identify factors at various levels of the social–ecological context that contribute to readiness to engage with child‐focussed DVA interventions, with the aim of improving understanding of how to enhance readiness, along with the types of interventions that may be acceptable to parents and children at different points along the readiness trajectory.

### 4.1 Parents’ readiness to engage with child‐focussed interventions

In line with the psychological readiness model (Cluss et al., 2006), our results emphasise the role of parental awareness of having experienced abuse from an intimate partner as a key internal factor that determines readiness. Our results extend the model by highlighting the particular importance of parents’ awareness of the impact of DVA on their children as an influence on readiness to engage in child‐focussed interventions, which appears to be mediated by a parent's ability to see beyond their own needs, enabling them to focus on those of their child/ren.

Women who have unresolved needs of their own may require a period of adult‐focussed support in order to see beyond their own needs to support their children to engage in therapeutic interventions. It is well documented that untreated parental mental health problems are associated with less optimal therapeutic progress and poorer outcomes among youth receiving mental health treatment (Beauchaine, Webster‐Stratton, & Reid, 2005; Pilowsky et al., 2008; Rishel et al., 2006). Visser et al., (2015) found that parent psychopathology in the context of DVA was indirectly linked to child adjustment through parent availability. They posit that that the emotional unavailability of traumatised parents may render it more difficult for them to adopt the perspective of their children, which they suggest is a key facilitator of benefit from trauma‐focussed interventions.

Social support, particularly from specialist DVA workers, emerged as a key facilitator of parental readiness to engage in a child‐focussed intervention, both through the mobilisation of practical assistance and help to recognise the impact of DVA on children's adjustment. Practitioners could play a role in helping parents understand *how* abuse occurring between caregivers, even if seemingly less severe and not directly witnessed, could have a detrimental impact on children, thus highlighting the need for a child‐focussed intervention. A large literature supports the critical influence of the therapeutic alliance on family engagement and retention (Elvins & Green, 2008). Families who experience a positive bond with the provider and a collaborative relationship for developing tasks and goals of treatment are more likely to remain engaged (Thompson, Bender, Lantry, & Flynn, 2007).

Specialist practitioners played a role in “priming” parents and children prior to their enrolment in an intervention, allowing them to address any practical barriers hindering attendance and to prepare parents and children in terms of what to expect. The function of this preparatory work is underscored by a review of approaches to family engagement and retention in child mental health programmes, which found improved engagement where providers explicitly addressed families’ practical (e.g. schedules, transportation) and psychological (e.g. family members’ resistance, beliefs about treatment process) barriers as they entered treatment (Ingoldsby, 2010). Our findings suggest that DVA workers undertake this preparatory engagement on an informal basis, although there may be merit in formalising this work by drawing on evidence regarding effective strategies shown to enhance programme uptake (Ingoldsby, 2010; Nock & Ferriter, 2005; Staudt, 2003). This emphasis on engagement would need to include a psychoeducational component focussing on the impact of DVA on children to highlight the possible need for intervention in the first place. This element would be important for parents who believe that their children may not have been adversely affected. There is also the need to address parental anxieties about the repercussions of their children sharing information about the abuse they have experienced; our results indicate that this is a key psychological barrier to engagement. In seeking help to secure their own and their children's physical safety, women report weighing the risk of ‘doing nothing’ against revealing abuse and risking the harm, abduction or removal of their children (Humphreys, Thiara, & Skamballis, 2011; Rhodes Cerulli, Dichter, & Barg, 2010; Rose et al., 2011). Our results suggest that these fears may continue as mothers seek help to support children's psychological recovery. In preparing mothers and children to take part in therapeutic interventions, it is imperative that both parties are fully informed about the limits of confidentiality and practitioners’ safeguarding duties so as to avoid a rupture of the therapeutic relationship if new information is disclosed and triggers a safeguarding response and to give mothers and children the opportunity to opt out of the intervention altogether. Addressing this issue directly may enable practitioners to reassure parents about the therapeutic benefits of children sharing their experiences in an attempt to minimise parental efforts to censor their children (McTavish et al., 2017). However, where there is concern that the sharing of information in the context of a therapeutic intervention may increase risk to children's safety, an intervention should not be offered.

### 4.2 Children's readiness to engage in therapeutic interventions

Priming work with children may also be necessary given that children who are coerced into participation may be unable to fully engage with the therapeutic processes that lead to positive change. Acknowledgement of DVA was a key dimension of children's readiness to take up an intervention. However, this depends in part on willingness to “break the secret” of abuse and the ability to discuss experiences. Therefore, children who have been coerced to attend an intervention may not be ready to share and may actively withdraw from the therapeutic process in an attempt to maintain some control.

A study of children's spontaneous disclosures of exposure to abuse in a psychoeducational programme found that children who were more ready to actively engage with an intervention were more likely to share their experiences, and that children who spontaneously disclosed their experiences reported greater benefits. However, this study found that nearly half of participating children were not ready to disclose their experiences in a group setting (Graham‐Bermann, Kulkarni, & Kanukollu, 2011). Indeed, a number of studies included in this synthesis highlight that the ability to articulate experiences and the willingness to share them may be intervention outcomes in and of themselves (Howarth, Moore, et al., 2016) and thus can be thought of as both a product of engagement and a precursor to benefit. It is perhaps unrealistic for children who have been chronically exposed to DVA to show clinically meaningful changes in well‐being and mental health outcomes following an intervention lasting for 8–12 weeks. Indeed “the group was a first and crucial step in a longer journey of healing” (p. 334) (Peled & Edleson, 1992). This implies that some children may benefit from repeated participation in group interventions or a period of psychoeducation prior to engagement to gain maximum benefit.

O'Doherty et al. (2014) argue that readiness for change should be measured as an intermediate outcome in trials of interventions for adult survivors of DVA, to explicate the causal mechanism through which an intervention may confer benefits or harms . Readiness for change could be used for stratification in randomisation (Ramsay et al., 2009) given that this characteristic along with others may have a bearing on both the acceptability and effectiveness of a particular intervention. Our results suggest that this thinking should be extended to the evaluation of child‐focussed interventions.

Not surprisingly, parental support was found to be a key in facilitating children's involvement in a therapeutic intervention. Encouraging a child to attend a programme may serve as implicit permission to share experiences with others (although not always as highlighted above), which as discussed above can facilitate more positive outcomes for children. Children identify their mothers as the person with whom they most often want to talk to about the abuse (Mullender et al., 2002), despite possible concerns that they may upset their parents (McGee, 2000; Mullender et al., 2002). Support from parents to engage in a therapeutic programme may signal to children that parents are emotionally available to have these conversations, while parents who do not actively facilitate their children's participation may convey, implicitly or explicitly, that they are not willing to talk about children's experiences of abuse. Meiser‐Stedman (2002) suggests that, for children to cope with traumatic stress, they need to form a coherent memory of the traumatic event represented in a verbal format (Meiser‐Stedman, 2002). This is a key aim of most DVA interventions; however, parents are critical in supporting this type of coping by communicating about the traumatic events with their child outside of the therapeutic context (Meiser‐Stedman, 2002). Where parents are supportive of participation, but are reticent to support children's sharing of experience, a longer period of priming work may be required.

### 4.3 Parent and child readiness as overlapping but distinct processes

Our results indicate that parent and child readiness are overlapping but distinct processes connected by parental ability and willingness to support engagement. Thus, a situation may arise whereby a child or young person is ready to take up an intervention, yet their parent is not ready to be involved or commit to assisting their child to attend. This is important to consider, given that mental health treatment studies show that initial attendance at an intervention is lowest when parents (rather than the adolescent or other family members) are unwilling to be involved in treatment (Santisteban, Suarez‐Morales, Robbins, & Szapocznik, 2006).

In the UK, there is consensus among professionals that children should be able to access interventions independently of their parents. This may be possible for an older adolescent or where another caregiver can support a child (Itzin et al., 2010); however, many of the interventions require the direct participation of the nonabusive parent. This is based on the rationale that enhancement of the parent–child relationship is a key pathway to children's recovery (Howarth, Moore, et al., 2016). Furthermore, our results suggest that as children make meaning of their experiences, they may raise questions such as “why did you stay?” that parents may find difficult to deal with if they are not adequately prepared. With this in mind, caution is required when offering a therapeutic intervention to children whose parent is unable or unwilling to engage; parents may feel alienated and resist involvement in supportive services altogether. In such circumstances, it may be preferable to work directly with parents to systematically address any perceived and modifiable barriers to engagement, which may also improve treatment outcomes.

### 4.4 Readiness as an ecologically nested construct

Similar to the readiness of female victims of DVA to engage in safety‐promoting behaviours (Heise, 1998, 2011), our results suggest that parent and child readiness to take up an intervention in the aftermath of DVA is determined by the confluence of individual, relationship and organisational factors.

In line with Cluss et al., (2006), we found relational factors such as the quality of interaction with a professional and support from a parent can influence internal factors such as awareness of abuse and in turn willingness to engage. We found that external factors also impacted readiness; however, the strongest theme to emerge was related to the organisational context of the agency offering support to the mother and child. Our findings suggest that organisational context can impact the ability of individual workers to deliver an intervention and to find the time needed to build rapport with parents and children; those who have reached a point of individual readiness may be less willing to take up an intervention that is offered by unskilled staff or by an organisation perceived as unable to place appropriate priority on children's recovery.

The role of organisational context is not explored by the psychological readiness model; however, this finding is consistent with the notion that complex interventions interact with the context in which they are delivered (Howarth, Devers, & Moore, 2016). Importantly, our findings suggest that this interaction may begin even before the intervention is delivered, at the point when an individual or family is weighing up whether to participate. In addition, programme structure (e.g. frequency and duration of sessions) and content, supervisory support, stability of funding, level of provider turnover and neighbourhood location may each influence family engagement, (Kumpfer, Alvarado, Smith, & Bellamy, 2002; McCurdy & Daro, 2001; McGuigan, Katzev, & Pratt, 2003). Furthermore, implementation researchers consistently cite the importance of practitioner, organisation and even community readiness for determining the quality of implementation and in turn service user engagement and eventual treatment outcomes (Fixsen, Naoom, Friedman, & Wallace, 2005). Several reviews of engagement and retention strategies in child and adolescent programmes concluded that only those strategies that address the broader ecological context of the family and the service delivery approach were effective in reducing the dropout rate (Ingoldsby, 2010; Staudt, 2003). An ecological perspective allows for the identification of multiple factors that may influence engagement and uptake of child‐focussed interventions and moves us away from viewing failure to engage as resulting exclusively from individual factors, such as motivation and resistance (Staudt, 2003). This may be particularly relevant when specialist DVA interventions are delivered by organisations lacking stable funding.

## 5 CONCLUSIONS

Three key findings emerge from this review of qualitative evidence exploring parent, child and professional experiences of delivering and receiving child‐focussed interventions following exposure to DVA. First, parent and child readiness is influenced by a complex interplay of individual, relationship and organisational factors, suggesting that individual readiness to take up child‐focussed interventions should be viewed through an ecological lens. Second, whilst the psychological readiness model can be used as a heuristic to describe parental readiness to engage in child‐focussed support, the specific process through which women become ready to engage in or facilitate interventions for children requires mothers to be aware of the impact of DVA on children and able to shift focus away from their own needs to those of their children, and thus differs from the process related to uptake of safety‐promoting behaviours. Third, our results highlight the distinct but interlinked processes through which parents and children reach a point of readiness to engage in an intervention aimed at improving child outcomes. These findings have some clear implications for both practice and research; however, it is pertinent to first consider the limitations of this study so that the reader can weigh these against one another.

## 6 LIMITATIONS

First, this synthesis included studies which evaluated the experiences of parents and children who had received an intervention and did not include studies focussing on the perspectives of parents and children, prior to uptake. Therefore, this study does not explore readiness from the vantage point of those who did not take up an intervention. The views of professionals provide some insight into the role that readiness plays; however, a more extensive exploration of the first‐hand accounts is required.

Second, the studies included in this review evaluated the experiences of delivering and receiving interventions when the risk of continuing abuse has reduced or ceased. Therefore, it is not possible to determine whether the findings presented here extend to readiness to take up different types of interventions, or to parents and children who are experiencing ongoing abuse. The studies reviewed in depth reported little on the readiness of the abusive party relating either to their own or the participation of other family members. As whole family models of intervention become more prevalent (Stanley & Humphreys, 2017), readiness of abusive parents to engage or allow engagement in child‐focussed interventions warrants further investigation.

Third, the evidence that is synthesised here is derived from studies where the majority of children were aged 4–12 and so is not necessarily generalisable to children of all ages. This is particularly relevant given the relatedness of parent and child readiness discussed here. Young people may have greater autonomy to access interventions in the absence of parental support and may value an intervention that is delivered independent of parents; indeed, guarantees of confidentiality from parents may be a prerequisite of older children's willingness to engage (Howarth, Moore, et al., 2016). Therefore, it is necessary to explore the construct of readiness and how this influences the appropriateness of particular interventions by the developmental stage.

## 7 CLINICAL AND RESEARCH IMPLICATIONS

This study offers insight into readiness of children and parents from an ecological perspective, which can help to guide the development of specific engagement strategies. Our results underscore the necessity for DVA interventions to address multilevel influences that determine readiness, rather than focusing solely on individual variables. The reasonably well‐developed evidence based on strategies to enhance family engagement in child and adolescent mental health interventions may serve as a foundation for this work, although the results presented here suggest that DVA engagement strategies should address the practical and emotional barriers that may prevent parents from focussing on their children's needs and also on the delivery of clear information for parents about DVA impact on children's well‐being. Our results also suggest that children who do not appear ready to take up an intervention may develop a sense of readiness through their participation; therefore, these children may benefit from access to an intervention on a second occasion or access to a sequence of interventions.

From a research perspective, there is a need to understand how initial parent and child readiness determines the acceptability and effectiveness of particular interventions; this will require mixed method trials of interventions to stratify by readiness at the point of randomisation (Ramsay et al., 2009). Readiness should also be measured as an intermediate outcome to examine its role in intervention (O'Doherty et al., 2014). Currently, there is a paucity of well‐validated tools to measure readiness.

Research on DVA interventions has largely ignored the roles of organisational and larger structural systems that shape interpersonal violence and the way that interventions are delivered (Howarth, Moore, et al., 2016). Our findings underscore the importance of the broader socioecological context in which interventions are offered in shaping whether the offer is taken up by parents and children. In addition to careful consideration of the acceptability of interventions to those receiving and delivering them, future intervention development should take account of the organisational and community contexts in which interventions are delivered, to enhance the likelihood of effective engagement.

## AUTHOR CONTRIBUTION

Theresa Moore oversaw and managed the review process, extracted constructs and worked on the synthesis. She drafted the manuscript and revised drafts and is joint first author and corresponding author. Emma Howarth provided a child development and domestic violence and abuse perspective, extracted constructs and worked on the synthesis. She drafted the manuscript and revised the drafts and is joint first author. Gene Feder provided domestic violence and health perspective and provided comments on the manuscript. He conceptualised the NIHR “Improving Outcomes for Children Exposed to Domestic Violence and Abuse,” research programme of which this was part. Harriet MacMillan provided a child health perspective and commented and shaped the manuscript. Ali Shaw oversaw the research, extracted construct, worked on the synthesis particularly in relationship to the ecological model, drafted the manuscript and commented on revisions to the draft. Ali also provided qualitative research expertise. Nicky Stanley provided a social work and child development perspective and commented and shaped the manuscript.

## CONFLICT OF INTEREST

Gene Feder, Emma Howarth, Harriet MacMillan, Theresa Moore, Ali Shaw and Nicky Stanley have no known conflicts of interest.
