# Supplementary material for: Towards an ecological understanding of readiness to engage with interventions for children exposed to domestic violence and abuse: Systematic review and qualitative synthesis of perspectives of children, parents and practitioners
Source: Health Soc Care Community. 2018 Jul 10;27(2):271–92. doi: 10.1111/hsc.12587 (PMC6392107; doi:10.1111/hsc.12587)
Supplement: Supplementary file 1 [file HSC-27-271-s001.docx]

# Supplementary material

## Example search strategy complete:

Database: MEDLINE: 08 04 2013

1 Child Welfare/ or Child, Preschool/ or Mother-Child Relations/ or Father-Child Relations/ or Child Behavior/ or "Child of Impaired Parents"/ or Child/ or Parent-Child Relations/ or Child Psychology/ or Child Reactive Disorders/ or Child Psychiatry/ or Adolescent Psychiatry/ or Adolescent Behavior/ or Adolescent/ or Adolescent Health Services/ or Adolescent Psychology/ or Adolescent Development/ (2312091)

(adolesc* or preadolesc* or pre-adolesc* or boy* or girl* or child* or infant* or preschool* or juvenil* or minors or school* or pediatri* or paediatri* or pubescen* or pre-pubescen* or puberty or student* or teen* or young or youth* or school* or high-school or "high school" or college or undergrad* or campus* or classroom*).mp. [mp=title, abstract, original title, name of substance word, subject heading word, keyword heading

word, protocol supplementary concept, rare disease supplementary concept, unique identifier] (3478462)

3 1 or 2 (3478462)

4 Domestic Violence/ or Spouse Abuse/ or Battered Women/ (10226)

5 (abuse* adj3 (wom*n or partner or spous* or m*n or wife or wives or husband*)).tw. (4405)

6 (battered adj3 (wom*n or partner or spous* or m*n or wife or wives or husband*)).tw. (650)

7 (violen* adj3 (wom*n or partner or spous* or m*n or wife or wives or husband*)).tw. (5174)

8 (marital adj3 (violen* or abuse*)).tw. (251)

9 (famil* adj3 (violen* or abuse*)).tw. (2397)

10 domestic violen*.tw. (3558)

11 (intimate adj3 partner adj3 (violen* or abuse*)).tw. (2536)

12 (interparental adj3 (violen* or abuse*)).tw. (71)

13 (violen* adj2 (home*1 or household*)).tw. (163)

14 (parent* adj3 (violen* or abuse*)).tw. (1516)

15 or/4-14 (17425)

16 (expose* or exposure).mp. (725218)

17 witnes*.mp. (13168)

18 growing up.tw. (1265)

19 ((child* or adolesc*) adj3 "living with").tw. (903)

20 ((child* or adolesc*) adj5 (violen* adj2 (home*1 or household*))).tw. (32)

21 ((child* or adolesc*) adj5 (domestic* adj2 violen*)).tw. (375)

22 or/16-21 (739605)

23 3 and 15 and 22 (1892)

This strategy was adapted for other databases and run:

Details of other databases searched:

Database: PsychINFO, Date of search <1806 to April Week 2 2013>

Database: Web of Science Social Sciences Citation Index. Date of search 09 04 2013

Database: Web of Science Conference Proceedings Citation Index- Social Science & Humanities

09 Date of search: 04 2013

Database: Social care online. Date of search 17 04 2013

topic="domestic violence" and (freetext="child* expose*" or freetext="child* witnes*")

Database: The Cochrane Library (All databases). Date of search 17 04 2013

Database: CINAHL on EBSCO. Date of search 17 04 2013

## Search strategy - Update search September 2015

Database: Ovid MEDLINE(R). Date of search <1946 to present> 23 September 2015

Database: PsycINFO. Date of searc. <1806 to September Week 3 2015>

Database: The Cochrane Library Date Run: 23/09/15

Database: Embase. Date of search <1974 to 2015 September 22>

Database: CINAHL Date of search: January 2013 to September 2015

## Search strategy - Update search June 2016

Database: PsychINFO, Date of search 11 June 2016

Database: Web of Science Social Sciences Citation Index. Date of search 11-05-2016

Database: Web of Science Conference Proceedings Citation Index- Social Science & Humanities

09 Date of search: 06-2016

Database: Social care online. Date of search 11-06 2016

topic="domestic violence" and (freetext="child* expose*" or freetext="child* witnes*")

Database: CINAHL on EBSCO. Date of search 16-06-2016

## Table S1 Details of included studies

| **Source Paper Country**  **Setting Respondents** | **Participant Type*, age, ethnicity, socioeconomic class, guardianship of children** | **Sample size and strategy** | **Data collection methods** | **Method of analysis/ Theoretical Approach** | **Description of intervention** | **Aim** |
| --- | --- | --- | --- | --- | --- | --- |
| 3828  Paris 1998  USA  **Setting:**Community:  **Respondents:** Children  Parents (both mothers and fathers)  Stakeholders | 8 families.  5 couples had children.  Children: n=4. 6 to 12 years.  Mothers: n=8  Fathers: male n=6  Families that wanted to stay together  The perpetrator parents had all previously attended a 23 week course for perpetrators of domestic violence (The Batterers’ Intervention Program- BIP). This attendance was ‘state mandated’.  Stakeholders:  Facilitators of the children’s and parent’s groups of RSVP. Providers of the BIP programme. | Children and parents who had attended the ‘field test’ of the RSVP programme. | Focus groups for children, and parents at the end of each group session to feedback to researchers about that session. Focus groups included interview guides. Each session also had a focus groups for facilitators. Observation of all child group sessions. | Constant comparison (Glaser and Strauss 1967) | RSVP programme. Group parallel psychoeducation for parents and children. Some whole family sessions. Time within sessions to practice new techniques. RSVP ran for 16 weeks, weekly meetings 90 minutes.  Children met in small groups. Presentation, followed by group activities and a period of free play. Parents met separately and together (abusing and non-abusing parents) for 20-30 minutes, then in large mixed group | “To develop and field test a structured multimodal family intervention strategy (RSVP) for use with small groups of families whose children have witnessed domestic violence.“ |
| 1763  Peled 1992  USA  **Setting:**Community  **Respondents:** Children  Parents (both mothers and fathers) Stakeholders  **Programme:**  Parents DAP (Domestic abuse project of Minneapolis) | Children: n=30 age: 4 to 12 years.  8 Living with both parents  2 living with grandparents  20 living with their mother  Working class, low to moderate income.  Mothers:16  Fathers: 5  Stakeholders: Group leaders and agency administration staff | All adult respondents attended DAP programme 1990 to 1991 | Interviews. In depth semi- structured. Plus observation of one group process (10 group sessions and 3 family sessions). Thematic guides. | Inductive content analysis Naturalistic research paradigm Lincoln and Guba 1985 | Group parallel psychoeducation intervention for children whose parents were in receipt of an intervention for DVA either as abuser or as victim. Children: “Gender-mixed,  age-specific (4-6,7-9,10-12) group psychoeducation activities were regularly offered to children of women and men.  in one of DAP's adult programs or to children who have parents in a similar program at a different agency.  At end of 10 weeks a single family session for parents and children to review material in child groups and recommend additional services.  Groups meet once a week for 10 weeks. Concurrent psychoeducational parenting groups were also available to parents. | “Analysis of one group program from the multiple perspectives of those who were involved. Attempted to understand both change inducing processes, and intended and unintended outcomes, rather than just measuring the achievement of expected goals.” |
| 3856  Peled 1998  USA  **Setting:** Community  **Respondents:** Children  Parents DAP (Domestic abuse project of Minneapolis) | Children: n=14. Mean age 11.3 yrs (10 to 13). 7 boys 7 girls. 5 living with both parents, 9 with mother only  Parents: n=12 Mothers  Comorbidities:  None of the children were physically abused. None were currently living exposed to DVA.  10/12 Mothers completed a ‘battered women’s’ group program  5/12 Fathers completed a ‘battering men’s’ group program. | Most families recruited through DAP and 2 from other community programmes. All children had witnessed domestic violence as reported by their mothers. | Informal conversational, interviewing. Interviews were loosely guided by a list of Categories of Desired Information. Children interviewed 3 to 6 times (Mean 4 X 1 hour interviews). | Inductive content analysis  Phenomenological inquiry Naturalistic enquiry  Lincoln and Giba 1985 & Patton 1990 | As above for Peled 1992 | One goal was to learn about ways in which children manage their exposure to violence.  ‘this study is about the experiences of preadolescent children of battered women who had participated in a therapeutic educational domestic group’ |
| 3860  Peled-1999  USA  **Setting:** Community  **Respondents:** Parents (both mothers and fathers)  Programme: DAP (Domestic abuse project of Minneapolis) | Children: n=204 10.4 (4 to 18) 61 (29.9%) of the children received services  Mothers; n=64  Fathers; n=41 | Contacted all eligible participants in a DAP (parents from 250 families) Participation of 42%. | Structured telephone interview, interview guide, multiple choice and open ended. This paper has data from the open-ended questions. | Inductive content analysis. Interviews were transcribed and coded. And clustered into 7 main themes. Then second analysis to 6 themes.  Patton 1990 | As above for Peled 1992? | Our main study question was “What are the factors that former adult clients of the agency identify as barriers to their child’s participation in and completion of available services?” |
| 3044  Humphreys 2006  UK  10 refuges and **Setting:** community settings  **Respondents:** Mothers  Stakeholders  “Talking with my mum” | No details in the paper: Participants as described in Humphries 2011 | Children and mothers and child workers interviewed separately by the researchers when they have completed the ‘Talking to my mum’ activity packs. | Focus groups of mothers and children and focus groups of with children’s workers in a refuge.  In the Humphries 2011 paper also interviews with mothers’ children and workers. | Action research. Stringer 1999.  No details were reported in this paper but Grounded research theory (Denzin and Lincoln) were reported in Humphreys 2011 | ‘Talking with my mum’ intervention developed at Colchester Women’s Aid Shelters and families in the community to improve communication between mothers and children in the aftermath of domestic violence. Additional centres for research were Refuge Essex (which includes all seven refuges in Essex), Panahghar (Refuge) in Coventry and Leicester, and Milton Keynes Women’s Aid. The research team was based at the University of Warwick | ‘To outline some of the abuse tactics that can damage mother-child relationships, together with their early impressions as to whether it may be possible to repair them through jointly focussed work. Some of the wider implications for social work practice are discussed.’ |
| 3045  Humphreys 2011  UK  **Setting:** Refuges  **Respondents:** Children  Mothers  Stakeholders  **Programme:**  Research project: “Talking with my mum” | Children:n=52 aged 5 to 16. 27 boys and 25 girls. (12 families were Asian).  Mothers: n=45  Families were living in DV refuges or at home but in receipt of DV services in UK (50% were living in a refuge).  Interviews with 15 workers. | 5 focus groups. Refection interviews with 45 mothers, and 52 children 27 boys and 25 girls. (12 families were Asian). Interviews with 15 workers | Focus groups of mothers and children and children’s workers in a refuge. Day workshops at the end of each ‘Action research cycle’ were taped and transcribed to identify themes from the data. Data from researchers’ ‘project log books’ were summarised. Data for families was looked at to identify key themes across families. | Grounded theory methods (Denzin and Lincoln 2003). | ‘Talking to my mum’  Mothers and children worked together on activities to improve their communication. The support of a trained refuge worker was available when needed. Children aged 5 to 16 years. The activities were developed and tested through ‘action research cycles’. Each cycle consisted of four to six months of implementation and continuous feedback, followed by two months of reflection and incorporation of feedback into the further development of materials.  Setting: 10 refuges and two specialist  voluntary sector community based counselling teams; and two voluntary sector domestic violence outreach projects. | To explore how communication between women and their children could be strengthened in the aftermath of DVA. What issues do workers, women and children identify as relevant to talk about? How do women and children evaluate materials developed to support their relationship in the aftermath of violence? |
| 0045  Kearny 2012  USA  **Setting:**Community  **Respondents:**  Parents mothers  Stakeholders  **Programme:** | Parents: n= 5 Mothers age 30 to 40 years Living in the Community with children age 5 to 12 years receiving a trauma focused psychiatric treatment . 5 informants (8 eligible, 6 consented and 1 dropped out of the programme)  Ethnicity: African American, Caucasian, and Hispanic. | Purposive sampling. And qualitative sampling of mothers whose children received trauma-focussed treatment in an ‘urban child guidance clinic’. The mothers were classified as ‘treatment resistant’ | Pre and post intervention interviews conducted by social workers (+ interviews with therapists). Maternal survey at end of intervention. Interviews with child therapists | No information about analytic method or theory. | Relational group work for mothers who had previously experience domestic violence whose children being treated for trauma. (relationally based psychoeducation grounded in psychodynamic and attachment theories), individual reflective feedback sessions, and ongoing brief phone contacts. This pilot intervention was designed as a supplement to the child’s treatment and not in place of any individual therapy that the mother may have been receiving. 6 weeks of Group work with 2 weeks of pre and post question sessions (8 weeks in total). | Evaluation of a multi-method pilot study of a relationally-based intervention with mothers of school-aged children receiving treatment for exposure to domestic violence. Two fold 1) to improve child outcomes. (Child functioning and progress in treatment). 2) to improve maternal functioning (psychological and behavioural*)* |
| 4414  Thompson-2011  USA  **Setting:** School  **Respondents:** Stakeholder  **Programme:** School based play therapy and psychoeduction | Stakeholder: First author of paper, White, middle class, woman. | Single stakeholder’s observations of four children who participated in the ‘Child centered group play therapy’. All were from a single school. Sessions happened at school. | Participant observation. Video and audiotaping of all 18 X 45 minute sessions, Observational notes made. Transcribed verbatim. | Retrospective case study using archival material. Observational notes were made during the viewing of each session. Each session was transcribed verbatim and transcripts read. Theory:  Erikson’s model of analytic induction | “Child centred group play therapy”. A group play intervention for children only. 18 sessions, delivered in School for 16 weeks. Four children three girls one boy. Age 6-7 years. Ethnicity: 2 African American one Latin American. | “The purpose of this qualitative case study is to illuminate the lived experiences of 4 young children between 6 and 7 years old who witnessed domestic violence while revealing the complex relationship between group process and stage development in their 18-week counseling group.” Abstract |
| 5690  Thompson 2011  USA  **Setting:** School  **Respondents:** Stakeholder – observation of children  **Programme:** | Stakeholder: First author of paper, White, middle class, woman. | Single stakeholder’s observations of four children who participated in the ‘Child centered group play therapy’. All were from a single school. Sessions happened at school. | As above for Thompson 2011 | As above for Thompson 2011 | “Child centred group play therapy”. A group play intervention for children only. Delivered in School.= for 16 weeks. Four children three girls one boy. Age 6-7 years. Ethnicity: 2 african American one Latin American. First nine sessions had 10 minutes of structured exercises and 35 minutes of group play therapy. Remaining nine sessions were 45 minutes of group play therapy. | A multimodal intervention with components for Adults and children. 16 weeks Responsible Steps Towards Violence Prevention Program (RSVP) (Page 34). Intervention included both abusing and non-abusing partner and children. The NA partner must have provided in writing the wish for the abusing parent to remain a part of the family. (Page 38 para 1). Children met in small groups with only children. Groups for parents were run concurrently |
| Jarman 2014  UK  **Setting:** Community  **Respondents:** Children  **Programme:** Drama therapy (Group) | Children: Boys age 7 to 9 years, all at least 6 months post separation from abusive parent. | 4 boys | Interview and observation. | Thematic analysis | Drama therapy for boys (15 week programme), plus parenting intervention for mothers. The drama therapy was based on the Embodiment, Projection and Role (EPR) model of drama therapy. This mirrors child development including physical, emotional and social development. This therapy allows a means to explore how child experiences can affect child development. | To “explore children’s experiences of drama therapy as treatment for those who have witnessed domestic abuse” |
| Ermentrout 2014  USA  **Setting:** Community  **Respondents:** Mothers Children Practitioners  **Programme:** MOVE Mothers Overcoming Violence through Empowerment | Women survivors of DVA who were involved in the criminal justice system as defendents. NB they were not perpetrators of DVA. Age 20 to 47 years, mean age 28 years. Single/separated/divorced n=8 (50%) In a relationship with abusive parent n=5 (63%).  Children aged 5 to 13 years.  Ethnicity African American, White (n=5, 31%, Other n=1 6%)  Practitioners delivering the MOVE programme. White (100%) females (90%)  Experience 1-5 years (n=3, 43%) 6 to 10 years (n=1, 14.3%) more than 10 years (n=3, 43%). | 18 women attended focus groups.  8 children focus groups or interview  9 practitioners focus groups. | Focus groups and interviews (for children) | Open coding approach using focus group guides and a representative transcript. Themes and sub themes identified using negative case analysis. Constant comparison was also used.  Coding was conducted iteratively | A 12 week programme of psychoeducation, and therapeutic parenting for mothers and a concurrent therapeutic support group for children. Specifically for justice-involved IPV survivors. | To evaluate a feasibility study of the community based – MOVE programme for mothers and children. |
| Cater 2014  Sweden  **Setting:** Community  **Respondents:** Children  **Programme:** Staircase | Children 4-19 years. Mean = 9 years | 29 children (15g 14b) No information on concurrent abuse. | Interviews. | Directed content analysis. Interview content was coded around participation of the children. Additional patterns in the material were coded subsequent to these. Contradictions were explicitly sought and used to stimulate alternative and more comprehensive interpretations. . | Individual, flexible counselling based on the ‘Staircase’ method which follows themes in a handbook. The therapy is not manualized and allows for flexibility. Children 15 years or older were referred directly and gave consent to attend. Children under 15 years, were consented when accompanied by their mother. Individual sessions were 30-60 minutes. Children received between 5-23 sessions. | “To explore and analyse processes of participation during counselling as described by children who had received a community based intervention for children exposed to IPV.” |

### Table S2 Translation of constructs: Children

| *2^nd^ Order constructs* | *Papers that include the 2^nd^ order construct* |
| --- | --- |
| **Personal readiness: contextual factors** |  |
| Adjustment to the ‘new reality’ in their lives | Peled 1998, Paris 1999 |
| Living in a refuge/shelter | Peled 1998 |
| Consequences of separation | Peled 1998 |
| Consequences of shelter living | Peled 1998 |
| Memory of abuse to siblings | Peled 1998 |
| Sadness at separation from father | Paris 1999,Peled 1998 |
| Inability to accept fathers abusive behaviour ‘reframing’ | Peled 1998 |
| Issues that children are arriving with e.g. sadness | Paris 1999, |
| Mother’s separation from father | Paris 1999, Peled 1998 |
| Ongoing vs. cessation of abuse | Peled 1998 |
| Priming children in advance of the intervention might be worthwhile | Humphreys 2011, Peled 1998, Ermentrout, Cater 2014 |
| Mismatch in readiness; children are ready but mothers are not | Humphreys 2011, Cater 2014 |
| **Personal readiness: willingness to break the secret** |  |
| Hesitancy to share what has happened | Thompson 2011, Cater 2014, Ermentrout 2014, Jarman 2013 |
| Sharing the experience/learning to share the experience | Peled 1992, Paris 1999, Peled 1998, |
| “I am not alone”: Beneficial; release of stress particularly beneficial to children who have not spoken of it before | Peled 1992 |
| Costs of remembering | Peled 1992, Humphreys 2011 |
| Comparing experiences could be beneficial; ’Realising things could have been worse’ but realising you had the worst experience could be harmful; ‘Shameful secret’ | Peled 1992 |
| Reducing shame and guilt | Peled 1992, Ermentrout 2014 |
| Depends on feeling safe | Peled 1992, Ermentrout 2014, Jarman 2013, Cater 2014 |
| Having someone to tell | Peled 1998, Cater 2014 |
| Not wanting to talk about the past | Peled 1998 |
| Not wanting to talk about their fathers | Peled 1998, Jarman 2013 |
| Acknowledging DVA had been a part of their lives | Peled 1998 |
| Readiness to talk | Paris 1999 |
| Safe, uninvolved adult was preferred | Cater 2014 |
| **Personal readiness: understanding and acknowledging DVA** |  |
| Learning the violence vocabulary | Peled 1992, Peled 1998 |
| Defining or labelling abuse | Peled 1992, Paris 1999, Peled 1998 |
| Attribution of responsibility for DVA | Peled 1992, Peled 1998 |
| ‘Abuse is not ok’: ‘de-normalising’ the abusive environment | Peled 1992, Peled 1998, |
| Need for support to acknowledge the abuse | Peled 1998, Jarman 2013 |
| Being asked explicitly about violence was a key to opening up | Cater 2014 |
| **Personal readiness: Initiating an intervention** |  |
| Mothers might not understand needs of child, while child does (Differential readiness) | Cater 2014 |
| Initiation driven by adult assumption of child needs | Cater 2014 |
| **Personal readiness: facilitators or motivators** |  |
| Child perception of benefits |  |
| Helping mothers and other children [altruism as motivation for joining in] | Humphreys 2011 |
| Children must be motivated if intervention is to work. | Cater 2014 |
| Positive affirmation strengthens self-esteem | Peled 1992 |
| Positive interaction with practitioner | Peled 1992 |
| Feeling special/value | Peled 1992 |
| Conflict resolution skills and strategies | Peled 1992 |
| Helping understand mum’s feelings | Humphreys 2011 |
| Helping understand my feelings | Humphreys 2011 |
| Enhancing communication (child mother) | Humphreys 2011 |
| Spending time with mum | Humphreys 2011 |
| Sharing feelings as a way of avoiding future distress (coping) | Peled 1998 |
| Spending time together (mother and child) was as important as activities themselves | Peled 1992, Humphreys 2006, Humphreys 2011 |
| Children must be motivated if intervention is to work. | Cater 2014 |
| Participation may be enhanced if children are involved in decision to take up intervention | Cater 2014 |
| Children need to trust their counsellors | Cater 2914 |
| **Derived benefits (stakeholder’s appraisal of benefit to child perspective)** |  |
| Ability to define abuse and understanding legitimacy of using abusive behaviour | Peled 1992, Thompson 2009 |
| Enhanced self esteem | Peled 1992, Thompson 2011 |
| Variation in outcomes for the child from ‘quite noticeable’ to ‘harder to identify. The intervention was ‘one step in a long journey’ | Peled 1992 |
| Sharing as therapy | Peled 1998, Thompson 2011 |
| Empathy for mothering situation | Peled 1998 |
| Expressing negative feelings is OK | Thompson 2009 , Thompson 2011 |
| Developing resistance | Thompson 2009, Thompson 2011 |
| Development of empathy | Thompson 2011 |
| Prosocial modelling | Thompson 2011 |
| Building resilience | Thompson 2011 |
| Safety planning | Thompson 2011 Peled 1992 |
| Learning to express angry feelings | Thompson 2011 |
| **Difficulties and tensions** |  |
| **Children’s perceptions of difficulties** |  |
| Psychoeducation around sexual abuse and prevention of was uncomfortable for the children | Peled 1992 |
| Stress to child caused by tension between perception of parent as abuser and love the child feels for that parent | Peled 1992, Peled 1998 |
| Need for tensions/stress to child to be managed by group leaders | Peled 1992 |
| Tensions are not always negative and may be uncomfortable (opening the door) for further work | Peled 1992, Peled 1998 |
| Confidentiality in group process led to tension of when was it permissible to ‘share’ with family or friends | Peled 1992 |
| Tension caused by changing or new family dynamics/rules | Peled 1998 |
| Seeing your caregiver (mother) as a victim and conflicting messages to child | Paris 1999 |
| Safety planning for the future may raise in a child’s mind possibility of future violence | Peled 1992 |
| **Costs and barriers to child** |  |
| Situational and practical missing school, television, or clubs | Peled 1992, Humphreys 2006 |
| Potential challenge of not being able to voice feelings with adults –including mother – Power dynamic | Humphreys 2011 |
| Not wanting to talk about past | Humphreys 2011, Peled 1998 |
| Not wanting to talk about their fathers | Humphreys 2011, Jarman 2013 |
| Children may be coached to not reveal things (Stakeholder perspective) | Ermentrout 2014 |
| Children may specifically not engage if they are there only because an adult has instructed them | Cater 2014 |
| **Factors of meeting in a group that enabled the therapeutic process** |  |
| Developing trust in the group | Peled 1992, |
| Having fun/making friends/ eating snacks | Peled 1992, Paris 1999, Peled 1998, Paris 1999 |
| Feeling safe | Peled 1992, Paris 1999 |
| Psychoeducation | Peled 1992 |
| Positive reinforcement by group leaders | Peled 1992 |
| Developing group norms and rules e.g. confidentiality | Peled 1992, Paris 1999 |
| ‘Ok not to talk’ feeling supported in the group and not compelled to talk | Peled 1992 |
| **Factors of meeting in a group that enabled the therapeutic process (Stakeholders perspective)** |  |
| Re-socialising, practising new behaviours, conflict resolution | Thompson 2009 ,Thompson 2011 |
| Developing group identity | Thompson 2009 , |
| Resistance and power struggles, testing limits | Paris 1999, Thompson 2009 |
| Disclosures to group members | Thompson 2009 |
| Children assume responsibility for group | Thompson 2009 , Thompson 2011 |
| Modelling of pro-social interactions | Thompson 2009 |
| Importance of group ending | Thompson 2009 , Thompson 2011 |
| Importance of group cohesion | Thompson 2009 , Thompson 2011 |
| **Child relationship with facilitator** |  |
| Modelling group leader interaction/behaviour | Peled 1992 |
| Potential challenge of not being able to voice feeling with adult because of a power dynamic | Humphreys 2011 |
| Building trust between practitioner and child, helps ‘Break the secret. | Cater 2014 |
| Therapeutic relationship can assist engagement | Cater 2014 |
| **Acceptability of interventions** |  |
| Parents appraisal of acceptability to child | Peled 1992 |
| Stakeholder appraisal of acceptability to child | Humphreys 2006, Paris 1999 |
| Child view of acceptability | Humphreys 2006, Humphreys 2011, Paris 1999 |
| Expectation of what it was going to be (child perception) | Humphreys 2006 |
| **Tailoring of intervention to child** |  |
| Variation in activities | Humphreys 2006, Paris 1999 |
| ***Locus of intervention/Recommendations*** |  |
| Father child relationship reducing confusion | Peled 1998, Thompson 2011 |
| Need to understand group process and how this will be manifest at different stages of development | Thompson 2009 ,Thompson 2011 |
| Length of intervention | Thompson 2009 , Thompson 2011 |
| Structuring of intervention: planned activities vs. unstructured play | Paris 1999, Thompson 2009 , Thompson 2011 |
| Need for parallel parent-child intervention | Paris 1999, Thompson 2009 |
| Setting within schools | Thompson 2011 |

Table S3 Translation of constructs - Parents

| *2^ND^ ORDER CONSTRUCTS* | *Papers that include the 2^nd^ order construct*  ****  ***** |
| --- | --- |
| **Readiness** |  |
| **1 They were in a DVA relationship** |  |
| Readiness of parents (fathers) to name and acknowledge DVA | Peled 1999*** |
| **2 DVA relationship has affected their child** |  |
| Acknowledgement of negative impact of DVA on child | Kearney 2012, Humphreys 2006, Peled 1999 |
| The way the intervention is presented to them feels appropriate | Peled 1999 |
| Perception of the reasoning behind the intervention | Peled 1999*** |
| Parents’ perception of child’s needs | Peled 1999 |
| ‘Protecting’ the child (prevention from participating) | Peled 1999** |
| Readiness to talk to their children about the past | Humphreys 2011 |
| Readiness to rebuild relationship with child | Humphreys 2006 |
| Parents need to know that their child is safe | Humphreys 2011 |
| Parents have a desire to get their child ‘back to normal’ | Humphreys 2011 |
| Primer preparatory work with parent on the impact of DVA on their child | Humphreys 2006, Humphreys 2011, Ermentrout 2014 |
| Mothers feel that children are emotionally OK to be in an intervention (safe and in a confidential space) | Ermentrout 2014 |
| **3 Able to see beyond their own needs to that of the child** |  |
| Parents fear their children’s disclosures | Ermentrout 2014 |
| Ready to see beyond own needs to those of their child | Kearney 2012, Humphreys 2011,Peled 1999***, Ermentrout 2014 |
| Their child’s opposition to attending | Peled 1999, Cater 2014 |
| **4 Practical aspects of readiness** |  |
| Timing/post crisis (practical aspects: baby-sitting, no time court cases; emotional fear uncertainty; time to reflect). | Humphreys 2006,Humphreys 2011, Ermentrout 2014 |
| **Benefits** |  |
| Improvement of reflective ability: comes through process of being in a group and seeing yourself through the eyes of others | Kearney 2012 |
| Seeing child as separate from self and abuser | Kearney 2012 |
| ‘I am not alone’/learning from each other (comes through process of being in a group) | Kearney 2012 |
| Normalisation of parents’ views of child behaviour, view of themselves (comes through the process of being in a group) | Kearney 2012 |
| Enhanced parenting development of sensitive plus realistic expectations of children | Kearney 2012 |
| Enhanced self-care ability to nurture the self | Kearney 2012 |
| Re-framing experience of abuse | Kearney 2012, Paris 1999 |
| Mastery of negative emotion and revelation of emotion leading to more effective problem solving | Kearney 2012 |
| Quality time together is beneficial of parent-child relationship | Humphreys 2006 |
| Learning or relearning to talk about the past | Humphreys 2006, Humphreys 2011 |
| Talking about the past with their children | Humphreys 2006, Humphreys 2011 |
| Developing a shared understanding between the parent and child of their situations. | Humphreys 2006, Humphreys 2011 |
| **Tensions for parent (where the parent intervention)** |  |
| Some felt a lack of role or a place for fathers | Peled 1999** |
| Addressing dynamics of power and control between perpetrator and non-abusing parent and ethos of feminism was a problem for some fathers | Paris 1999** |
| Parents may need access to additional support in coming to terms with what the children tell them (after the children receive an intervention) | Peled 1992,Humphreys 2006 |
| Maternal stress from the child’s re-framing and/or re-evaluating abuse and criticising mother. | Peled 1992 |
| The new capacity for the child to express feelings was bitter sweet | Peled 1992 |
| The intervention must be presented in a way that parents find acceptable (readiness of parents: priming for parents) | Peled 1999, Ermentrout 2014 |
| Practicalities: Time missing television, leaving work early, babysitting siblings, waiting for an opening in a group, transportation, time, those with shared custody had limited time or control over child’s activities and restricted time with child (Perpetrator) | Peled 1992,Humphreys 2006, Peled 1999 |
| Interference by co-parents may prevent child attending | Ermentrout 2014 |
| Perception of the intervention (philosophy/feminism) | Peled 1999*** |
| Child’s opposition to attending | Peled 1999 |
| Focus on confidentiality in group processes for children can lead to a loss of sense of control for the mother | Peled 1992 |
| **Process through which changes were effected** |  |
| ‘I am not alone’/learning from each other comes through process of being in a group | Kearney 2012 |
| the group sessions become a holding place (safe psychological space) | Kearney 2012 |
| mixed versus single gender groups (safe psychological space) | Paris 1999 |
| ‘Safe space’ Promotes disclosure and honesty and bonding and safe expression of emotion | Paris 1999 Humphreys 2006 |
| ‘Good grandmothering’ overseeing parenting | Kearney 2012 |
| Individual and group sessions valued for mothers | Kearney 2012 |
| Enhanced self-care/nurturing self | Kearney 2012 |
| ‘More is more’: Parents wanted more of the interventions, longer programmes and longer individual sessions | Kearney 2012, Paris 1999 |
| Strengths based approach/ individualised | Humphreys 2006 |
| Mother child relationship must be a focus of the intervention | Humphreys 2006 |
| Re-framing experience of abuse | Kearney 2012, Paris 1999 |
| Focus on ‘mother’ and ‘woman’ roles | Kearney 2012 |
| **Parent view of benefit to child of empowerment** |  |
| Children have confidence to challenge abusive behaviour in their family | Peled 1992 |
| Increased emotional expressiveness of child | Peled 1992 |
| Correct attribution of blame for violence | Peled 1992 |
| Resilience is noticed for example child’s knowledge of safety planning | Peled 1992 |
| Child’s awareness of measures around sexual abuse | Peled 1992 |
|  |  |

** Includes views of perpetrator parent as well as non-perpetrator parent

*** Views of perpetrator parent

### Table S4 Translation of constructs – Practitioners

| *Dimensions of 2^ND^ ORDER CONSTRUCTS Stakeholder* | *Papers that include the 2^nd^ order construct*  ***  **** |
| --- | --- |
| **Personal readiness: Child as reported by stakeholder**  **Stakeholder view of child readiness** |  |
| Child readiness | Peled 1992 Humphreys 2006 |
| Adjustment to the ‘new reality’ in their lives | Peled 1998, Thompson 2011 , Paris 1999 |
| Issues that children are arriving with e.g. sadness | Paris 1999, Thompson 2011 |
| Children need time to develop trust in the group | Thompson 2011, Ermentrout 2014 |
| **Personal willingness: willingness to break the secret** |  |
| Hesitancy to share what has happened | Thompson 2011, Ermentrout 2014 |
| Sharing the experience/learning to share the experience | Peled 1992, Paris 1999, Thompson 2009 Thompson 2011 |
| Having someone to tell | Thompson 2009 |
| **Personal readiness: understanding and acknowledging DVA** |  |
| Learning the violence vocabulary | Peled 1992, |
| Defining or labelling abuse | Peled 1992, Paris 1999 |
| Attribution of responsibility for DVA | Peled 1992, |
| ‘Abuse is not ok’ – ‘de-normalising’ the abusive environment | Peled 1992, Thompson 2011 |
| **Personal readiness: mother as reported by stakeholder**  **Stakeholder view of mother readiness** |  |
| Situational readiness: families must not be in crisis | Humphreys 2006; Humphreys 2011 |
| Safe: families must be away from abuser | Humphreys 2011 |
| Parents must acknowledge effect of DVA on children | Humphreys 2011 |
| Parents must have insight to address child needs | Humphreys 2011 |
| Priming of mothers before introducing the intervention may be worthwhile | Peled 1992 Humphreys 2011, Ermentrout 2014 |
| **Organisational readiness** |  |
| Quality of mother child relationship | Humphreys 2011 |
| Facilitation of intervention (timing and readiness of mothers) by practitioners | Humphreys 2011 |
| Organisational readiness | Humphreys 2011 |
| Integrating intervention with everyday practice (in refuge) |  |
| **Stakeholder/organisational readiness** |  |
| Worker readiness | Humphreys 2011 |
| Skills of working with women and children | Humphreys 2011, Ermentrout 2014 |
| Must engender trust in the child (children’s perspective) | Cater 2014 |
| Adaptability / flexibility of the programme helped practitioners deliver it | Cater 2013, Ermentrout 2014 |
| ***Derived benefits from intervention; stakeholder perceptions of benefits for child*** |  |
| Mental health symptoms | Kearney 2012 |
| Reductions of feelings ‘I am not alone’ | Paris 1999 |
| Assisting them in ‘breaking the secret’ | Paris 1999 |
| Ability to define abuse and understanding legitimacy of using abusive behaviour | Peled 1992, Thompson 2009 |
| Enhanced self esteem | Peled 1992, Thompson 2011 |
| Variation in outcomes from ‘quite noticeable’ to ‘harder to identify’ – the intervention was ‘one step in a long journey’ | Peled 1992 |
| Sharing as therapy | Thompson 2011 |
| Expressing negative feelings is OK | Thompson 2009 Thompson 2011 |
| Developing resistance | Thompson 2009 Thompson 2011 |
| Development of empathy | Thompson 2011 |
| Prosocial modelling (having a go at doing something nicely) | Thompson 2011 |
| Building resilience | Thompson 2011 |
| Safety planning | Thompson 2011 Peled 1992 |
| Learning to express angry feelings | Thompson 2011 |
| ***Derived benefits from intervention: Stakeholders views of benefits for parent*** |  |
| Improved engagement with services (mother) | Kearney 2012, |
| Treatment compliance (mother) | Kearney 2012 |
| More time together with child (mother) | Paris 1999 |
| Enhanced communication with partner (Both parents) | Paris 1999 |
| Change and growth (both partners) | Paris 1999 |
| ***Experiences of intervention: therapeutic relationship (with practitioners)*** |  |
| Transition from hesitancy to engagement (mothers) | Kearney 2012 |
| Adequate communication between parents and group leaders | Peled 1992 |
| Creating trust/rapport for the therapeutic relationship trust | Humphreys 2011 Paris 1999 |
| **Group process** |  |
| Re-socialising, practising new behaviours, conflict resolution (children) | Thompson 2009 ,Thompson 2011 |
| Developing group identity (children) | Thompson 2009 |
| Resistance and power struggles, testing limits (children) | Paris 1999, Thompson 2009 |
| Disclosures to group members (children) | Thompson 2009 |
| Children assume responsibility for group (children) | Thompson 2009 Thompson 2011 |
| Modelling of pro-social interactions (children) | Thompson 2009 |
| Importance of group ending (children) | Thompson 2009, Thompson 2011 |
| Importance of group cohesion (children) | Thompson 2009 Thompson 2011 |
| Gender mix of groups (adults) | Paris 1999 |
| Mix of abusing and not abusing participants (adults) | Paris 1999 |
| Pro-social modelling for children in groups. (children) | Paris 1999 |
| Challenge children’s behaviour (children) | Paris 1999 |
| Peer interactions (children) | Paris 1999, Ermentrout 2014 |
| Group cohesiveness (children) | Paris 1999 |
| Catharsis, expression of negative emotions (children) | Paris 1999 |
| Adults – provide a safe and supportive environment (adults) | Paris 1999 |
| More time in groups to practice (adults) | Paris 1999 |
| Time required in group to practice (adults) | Paris 1999 |
| Catharsis (children | Paris 1999 |
| Ethos of group | Paris 1999 |
| **Barriers and facilitators** |  |
| Crises in the family situation | Humphreys 2011 |
| Culture e.g. fear of family shaming | Humphreys 2011 |
| Quality of mother child relationship | Humphreys 2011 |
| Power dynamic between adults and children. Child may opt to not engage with the intervention | Cater 2013 |
| **Tensions and costs** |  |
| Reconciling abuser as father (for children) | Paris 1999 |
| Ongoing safety issues (for children) | Paris 1999 |
| When doing whole family work, group work with abusing and non-abusing families can reinforce power imbalances. | Paris 1999 |
| **Limitation to intervention** |  |
| Group intervention is just one part of the process ‘one step on a long journey’ | Peled 1992 |
| **Tailoring of interventions** |  |
| Tailor to child experience as therapeutic process | Peled 1992 Paris 1999 |
| Presence of child abuse | Peled 1992 |
| Involvement versus non-involvement of parent | Peled 1992 |
| **Content of parent/ adult intervention; directed at abusive parent** |  |
| Alcohol | Paris 1999 |
| Anger, more constructive management without enacting violence | Paris 1999 |
| Gender stereotyping | Paris 1999 |
| Power dynamics | Paris 1999 |
| **Acceptability** |  |
| Child | Humphreys 2006 |
| Stakeholder | Humphreys 2006; |
| **Locus of intervention (recommendations)** |  |
| Father child relationship reducing confusion | Thompson 2011 |
| Need to understand group process and how this will be manifest at different stages of development | Thompson 2009, Thompson 2011 |
| Length of intervention | Thompson 2009, Thompson 2011 |
| Structuring of intervention – planned activities vs unstructured play | Paris 1999, Thompson 2009, Thompson 2011 |
| Need for parallel parent-child intervention | Paris 1999, Thompson 2009 |
| Setting School | Thompson 2011 |
